# Decoding UTROSCT heterogeneity: systematic clinicopathological evaluation combined with molecular profiling

**DOI:** 10.1002/2056-4538.70055

**Published:** 2025-11-29

**Authors:** Jing Yang, Jinku Zhang, Jinmei Li, Yan Liu, Yuxiang Wang, Ajin Hu, Congrong Liu

**Affiliations:** ^1^ Department of Pathology, Peking University Third Hospital, School of Basic Medical Sciences Peking University Beijing PR China; ^2^ Department of Pathology First Central Hospital of Baoding Baoding PR China; ^3^ Key Laboratory of Molecular Pathology and Early Diagnosis of Tumor in Hebei Province First Central Hospital of Baoding Baoding PR China

**Keywords:** UTROSCT, prognosis, copy number variation, SWI/SNF, SMARCB1

## Abstract

Uterine tumor resembling ovarian sex cord tumor (UTROSCT) constitutes an exceptionally rare histological subset of uterine mesenchymal neoplasms. While most cases have benign clinical behavior, a subset of UTROSCTs exhibits clinically aggressive behavior characterized by recurrence and metastasis. Here, we present a cohort of 25 UTROSCT cases molecularly confirmed by recurrent fusion gene detection, including *ESR1::NCOA3* (*n* = 12), *GREB1::NCOA1* (*n* = 6), *ESR1::NCOA2* (*n* = 3), *GREB1::NCOA2* (*n* = 2), *GREB1::SS18* (*n* = 1), and *GREB1::CTNNB1* (*n* = 1). Notably, six cases (6/25, 24%) demonstrated recurrence/metastasis: two cases showed intrauterine recurrence (harboring *ESR1::NCOA3* and *GREB1::NCOA1* fusions), while four developed extrauterine metastases (carrying *ESR1::NCOA3*, *ESR1::NCOA2, GREB1::NCOA1,* and *GREB1::NCOA2* fusions), with one fatality. To dissect the biological basis of UTROSCT aggressiveness, we performed integrated clinicopathologic, immunohistochemical, and molecular profiling. Multivariate analysis identified tumor size >5 cm, FIGO stage IB, and lymphovascular space invasion (LVSI) as independent predictors of recurrence/metastasis, whereas histologic features, proliferation index, and fusion gene subtypes lacked prognostic significance. Multi‐omics analysis of primary versus metastatic tumors revealed striking copy number variations (CNVs) exclusively in metastatic lesions. Specifically, heterozygous losses of *SMARCB1* (2/4 metastatic cases) and *ATRX* (1/4 metastatic cases) were identified; both play critical roles in chromatin remodeling. These genetic alterations were conspicuously absent in primary tumors, suggesting their potential role in metastatic progression. Our findings represent the first demonstration of CNV‐driven oncogenic evolution in UTROSCTs, particularly implicating SWI/SNF complex dysregulation in metastatic competence.

## Introduction

Uterine tumor resembling ovarian sex cord tumor (UTROSCT) is a rare uterine mesenchymal neoplasm characterized by distinctive sex cord‐like architectural patterns and polyphenotypic immunohistochemical profiles. Initially described by Clement and Scully in 1976 as a morphologic counterpart to ovarian sex cord tumors, UTROSCTs are typically classified as benign tumors with indolent clinical courses [1]. However, sporadic reports of recurrence, metastasis, and rare fatal outcomes [2, 3, 4, 5, 6, 7] highlight their biological heterogeneity, complicating therapeutic decision‐making – particularly in balancing disease control with organ‐preserving strategies. Polypectomy remains the fertility‐sparing standard of care for most patients, yet persistent risks of recurrence and metastasis in subsets underscore the urgent need for refined prognostic stratification tools to enable individualized management.

Despite advancements in understanding UTROSCT, consensus on prognostic criteria remains elusive. While certain morphologic features – such as large tumor size, cytologic atypia, high mitotic indices, infiltrative growth, lymphovascular space invasion (LVSI), and necrosis – have been proposed as indicators of aggressive behavior [8, 9], conflicting evidence exists across studies [8]. Emerging molecular insights have identified recurrent fusion genes involving *NCOA1‐3, GREB1*, and *ESR1* rearrangements, with preliminary suggestions of genotype–phenotype correlations [10, 11]. However, limited clinical data on recurrent/metastatic cases have hindered the establishment of definitive risk factors, and the molecular drivers of adverse outcomes remain poorly defined.

The SWItch Sucrose Non‐Fermentable (SWI/SNF) chromatin remodeling complex acts as a pivotal epigenetic regulator, dynamically orchestrating chromatin structure to facilitate DNA replication, transcription, repair, and recombination. By modulating chromatin compaction and decompaction, this complex ensures precise spatiotemporal control over genomic accessibility [12]. Notably, over 20% of solid and hematologic malignancies in both pediatric and adult populations harbor deficiencies in SWI/SNF complex function [13, 14]. In mammals, the SWI/SNF complex comprises 12–15 subunits, with its functional core formed by an ATPase subunit (either *SMARCA4*/BRG1 or *SMARCA2*/BRM) and three additional subunits (*SMARCB1*/INI1, *SMARCC1*, *SMARCC2*). Among these, *SMARCB1*/INI1 emerges as a frequent target of inactivating mutations in soft tissue tumors, where its loss disrupts critical tumor suppressor pathways. Despite its established role in oncogenesis, the specific contribution of SWI/SNF dysfunction to the pathogenesis and clinical progression of UTROSCT remains underexplored.

To address these gaps, we conducted a comprehensive analysis of 25 UTROSCT cases molecularly confirmed by fusion gene detection. Through integrated evaluation of clinical, histopathologic, immunohistochemical, and molecular features, we identified tumor size, FIGO stage, and LVSI as significant predictors of recurrence and metastasis. Notably, other clinicopathologic parameters showed no prognostic association. To dissect the metastatic mechanism, we performed multi‐omic next‐generation sequencing (NGS) of matched primary and metastatic tumors, comparing single nucleotide variations (SNVs), indels, copy number variations (CNVs), and fusion landscapes. Strikingly, metastatic tumors exclusively exhibited heterozygous loss of key tumor suppressor genes, implicating CNVs as critical determinants of aggressive behavior.

## Materials and methods

### Patient selection

Twenty‐four cases of UTROSCT were selected from Peking University Third Hospital from 2010 to 2024, and one case was obtained from First Central Hospital of Baoding. Each case was meticulously reviewed by three experienced pathologists (JY, YW, and CL), leading to a unanimous histologic consensus in all instances. The diagnosis of UTROSCT in these 25 cases was further confirmed genetically by the presence of recurrent fusion genes, which are characteristic of this tumor type. Notably, cases exhibiting typical morphologic and immunohistochemical features of UTROSCT but lacking detectable recurrent fusion genes were excluded from our study to ensure diagnostic accuracy. Formalin‐fixed paraffin‐embedded (FFPE) tissues were available for all 25 cases. Clinicopathologic data were obtained from medical records and pathology reports. Follow‐up information was obtained from online electronic medical records or through the telephone. This study was approved by the Biomedical Ethics Committee of Peking University Third Hospital (M20250467).

### Immunohistochemistry

FFPE specimens were sectioned at 4 μm thickness and mounted on glass slides. Sections were initially incubated at 60°C for 2 h to enhance tissue adhesion. Deparaffinization was achieved through sequential immersion in xylene followed by a graded ethanol series for rehydration. Antigen retrieval was conducted in a pressure cooker using 1 l of EDTA buffer (1 mmol/l, pH 9.0) at boiling temperature for optimal epitope exposure. Endogenous peroxidase activity was quenched by incubating sections in a 0.3% hydrogen peroxide solution for 10 min, followed by thorough rinsing in phosphate‐buffered saline (PBS) for 2 min. Sections were blocked with appropriate serum prior to primary antibody application. After 30 min incubation at room temperature, unbound antibodies were removed by three consecutive washes in PBS. Visualization was achieved using species‐specific horseradish peroxidase‐conjugated secondary antibodies (Anbiping Pharmaceutical Technology, Guangzhou, PR China) with a 20‐min room temperature incubation. Immunoreactivity was detected using 3,3'‐diaminobenzidine tetra hydrochloride (DAB) chromogen substrate (Anbiping Pharmaceutical Technology) applied for 5 min at room temperature, followed by hematoxylin counterstaining and standard dehydration procedures prior to coverslipping.

The primary antibodies used in this study included ER (ab16660, 1/250, Abcam), PR (PgR636, 1/100, Dako), α‐inhibin (GTX40215, 1/100, GeneTex), Calretinin (DAK‐Calret 1, 1/10, Dako), WT1 (6F‐H2, 1/100, Abnova), SF‐1 (N1665, 1/100, Novus), CD10 (56C6, 1/50, Abcam), Desmin (D33, 1/20, GeneTex), SMA (ab184705, 1/100, Abcam), h‐caldesmon (EP19, 1/100, HZbscience), CK (AE1/AE3, 1/100, ZSGB‐BIO), Ki‐67 (ab15580, 1/100, Abcam), INI1 (ab192864, 1/1000, Abcam), BRG1 (ab110641,1/100, Abcam), BRM (ab240648, 1/1000, Abcam) and ATRX (ab188027, 1/200, Abcam).

### Fluorescence *in situ* hybridization

Fluorescence *in situ* hybridization (FISH) analyses were performed on 4‐μm FFPE slides. Standard staining procedures encompassed deparaffinization, pretreatment, and the hybridization of probes onto unstained slides. After an overnight incubation, the slides underwent rinsing and were stained with 4',6‐diamidino‐2‐phenylindole (DAPI). Following mounting, they were examined under a fluorescence microscope (Zeiss Axioplan, Germany).

Break apart FISH probes for *ESR1, GREB1, NCOA1, NCOA2, and NCOA3* were all acquired from Anbiping Pharmaceutical Technology. The positive cutoff criterion was established as the presence of a split pattern and/or a single signal in at least 20% of the tumor cells.

A dual‐color probe with *SMARCB1* as the target (located at 22q11, red fluorochrome) and *EWSR1* as the control (located at 22q12, green fluorochrome) was also purchased from Anbiping Pharmaceutical Technology. A cutoff value for heterozygous deletion of *SMARCB1* was set at 30% of the cells with one signal for the *SMARCB1* probe and one or two signals for the control probe.

### DNA based next‐generation sequencing

DNA was extracted from FFPE samples using the QIAGEN QIAamp DNA FFPE Tissue Kit (Catalog No. 56404). DNA concentration was subsequently measured by Qubit™ dsDNA HS Assay Kit. The pre‐capture libraries were enriched for the specific genes of interest through a method based on hybridization capture. The specially designed and biotinylated probes that span gene regions of interest targeted by the assay were hybridized to the libraries. Following hybridization and incubation, streptavidin magnetic beads were employed to isolate the probes and hybridized targeted libraries from the nontargeted ones. After washing, amplification, and purification, the targeted enriched libraries were obtained. DNA fragmentation was performed using a Covaris M220 Focused‐ultrasonicator (Woburn, MA, USA). Quantify the enriched library with a Qubit™ dsDNA HS Assay Kit. The main band of library fragments should be within 200–400 bp, and be devoid of adaptors and large fragment contamination. DNA libraries were captured by Oncology Multi‐Gene Variant Assay, which covers 1,021 tumor‐related genes (supplementary material, Table S1). Subsequently, single‐stranded DNA nanoballs (DNBs) were created and loaded to a flow cell. The DNBs are sequenced using combinatorial probe anchor synthesis‐based sequencing technology on the MGI sequencing platform (DNBSEQ‐G50/G400) with related sequencing set (FCS/FCL PE100). Upon completion of high‐throughput sequencing, the Geneplus Box is utilized to analyze the sequencing data. The sequence information obtained is transmitted to the Geneplus Box data analysis and management system, which facilitates the comparison and analysis of the raw data. This streamlined process enables the automatic generation of reports from the downloading of raw data to the final output, ensuring efficiency and accuracy in data analysis.

### RNA based next‐generation sequencing

Total RNA was digested with the TIANSeq rRNA Depletion Kit (Tiangen Biotech, PR China) and the quality of purified RNA was assessed by the Agilent 2100 bioanalyzer (Agilent, WA, USA). Subsequently, the constructed library underwent pair‐end sequencing to generate 100‐bp reads on the DNBSEQ‐T7RS sequencing platform (Geneplus, PR China), following the manufacturer's guidance.

The constructed library was then paired‐end sequenced in 100‐bp lengths with the DNBSEQ‐T7RS sequencing platform (Geneplus). Raw data from NGS was then filtered to remove low‐quality reads and adaptor sequences. Furthermore, the reads were aligned to the reference human genome (hg19) using STAR software [15]. The gene list for RNA sequencing can be viewed in supplementary material, Table S2.

### Statistical analysis

Statistical analysis was conducted utilizing SPSS version 20.0 software. Univariate analysis of categorical variables was conducted, employing the Pearson's *χ* ^2^ test and Fisher's exact test. All tests were two‐tailed and statistical significance was defined as *p* < 0.05.

## Results

### Clinicopathological characteristics of UTROSCT

The clinicopathological characteristics of 25 UTROSCTs are summarized in Table 1. The patient cohort demonstrated a broad age range at initial diagnosis (mean 44 years; range 19–71 years) with comprehensive clinical follow‐up (median 46.2 months; range 6–161 months). Favorable outcomes were achieved in 19 patients (76%) who remained disease‐free after surgical resection while 6 patients (24%) experienced disease progression including 2 with uterine recurrence and 4 with extrauterine metastases.

**Table 1:** Clinicopathologic features and fusion genes of UTROSCT cases

Case no.		Fusion gene	Age (years)	Tumor size (cm)	Specimen type	FIGO stage	Microscopic tumor margins	Tumor architecture	Cytomorphology	Nucleoli	Cellular atypia	Mitosis (/10HPF)	LVSI	Necrosis	Follow up (duration in months)
**Cases without recurrence/metastasis**															
1		*ESR1::NCOA3*	70	2 × 1.5 × 1	Polypectomy	IA	Infiltrative	Diffuse, papillary	Spindle and epithelioid cells	Absent	Mild	≤1	Absent	Absent	NED (49 m)
2		*ESR1::NCOA3*	33	4 × 3 × 3	Polypectomy	IA	Infiltrative	Sex cord, nested	Epithelioid cells	Present	Mild	≤1	Absent	Absent	NED (49 m)
3		*ESR1::NCOA3*	29	2 × 1.5 × 1	Polypectomy	IA	Infiltrative	Sex cord, retiform	Spindle and epithelioid cells	Present	Mild	≤1	Absent	Absent	NED (42 m)
4		*ESR1::NCOA3*	23	2 × 1.6 × 1.5	Polypectomy	IA	Infiltrative	Sex cord, diffuse, whorled	Spindle and epithelioid cells, rhabdoid cells	Absent	Mild	≤1	Absent	Absent	NED (43 m)
5		*ESR1::NCOA3*	32	2 × 1 × 1	Polypectomy	IA	Infiltrative	Sex cord, diffuse, adenosarcoma‐like	Epithelioid cells	Present	Mild	≤1	Absent	Absent	NED (39 m)
6		*ESR1::NCOA3*	28	4.4 × 3 × 2	Polypectomy	IA	Infiltrative	Sex cord, diffuse, whorled	Spindle and epithelioid cells	Present	Mild	≤1	Absent	Absent	NED (45 m)
7		*ESR1::NCOA3*	50	1 × 1 × 0.5	THBSO	IA	Infiltrative	Sex cord, diffuse, nested	Epithelioid cells	Present	Mild	1–2	Absent	Absent	NED (16 m)
8		*ESR1::NCOA3*	57	15 × 11.3 × 11.5	THBSO	IB	Infiltrative	Sex cord, diffuse, nested	Epithelioid cells	Absent	Mild	3–5	Absent	Absent	NED (26 m)
9		*ESR1::NCOA3*	39	3.5 × 3.2 × 3.3	Polypectomy	IA	Infiltrative	Sex cord, diffuse, nested	Epithelioid cells	Present	Mild	1–2	Absent	Absent	NED (12 m)
10		*ESR1::NCOA3*	25	2.5 × 1 × 1	Polypectomy	IA	Circumscribed	Sex cord, diffuse, nested	Epithelioid cells	Present	Mild	≤1	Absent	Absent	NED (38 m)
11		*ESR1::NCOA2*	34	5.3 × 3 × 3	Polypectomy	IB	Infiltrative	Sex cord, diffuse, nested	Epithelioid cells	Present	Mild	≤1	Absent	Absent	NED (24 m)
12		*ESR1::NCOA2*	27	1.4 × 1.1 × 1	Polypectomy	IA	Infiltrative	Sex cord, nested, retiform	Spindle and epithelioid cells	Present	Moderate	≤1	Absent	Absent	NED (22 m)
13		*GREB1::NCOA1*	55	4 × 2 × 1.2	THBSO	IA	Infiltrative	Sex cord, diffuse, nested	Epithelioid cells	Present	Mild	≤1	absent	absent	NED (97 m)
14		*GREB1::NCOA1*	66	4.6 × 4 × 2	THBSO	IA	infiltrative	sex cord, diffuse, nested, whorled, papillary	spindle and epithelioid cells	present	mild	≤1	Absent	Absent	NED (16 m)
15		*GREB1::NCOA1*	65	4 × 3.5 × 2	THBSO	IA	Circumscribed	Sex cord, nested	Epithelioid cells	Present	Mild	≤1	Absent	Absent	NED (42 m)
16		*GREB1::NCOA1*	71	5.5 × 4 × 2	THBSO	IB	Infiltrative	Sex cord, diffuse, nested, whorled	Spindle and epithelioid cells	Present	Moderate	1–3	Absent	Present	NED (62 m)
17		*GREB1::NCOA2*	63	3 × 2 × 1.5	THBSO	IA	Infiltrative	Diffuse, nested	Epithelioid cells	Present	Mild	3–5	Absent	Absent	NED (76 m)
18		*GREB1::SS18*	44	3 × 2.5 × 2	THBSO	IA	Circumscribed	Sex cord, diffuse	Epithelioid cells	Present	Mild	9–12	Absent	Present	NED (6 m)
19		*GREB1::CTNNB1*	37	6 × 5 × 5	TH	IB	Infiltrative	Sex cord, diffuse	Epithelioid cells	Present	Mild	3–5	Absent	Absent	NED (24 m)
**Uterine recurrence cases**															
20		*ESR1::NCOA3*	19	6.5 × 5.6 × 5.3	Polypectomy, and TH after recurrence	IB	Infiltrative	Diffuse, nested	Spindle and epithelioid cells	Present	Moderate	≤1	Absent	Present	Recurrence 6 m after polypectomy, NED (32 m) after TH
21		*GREB1::NCOA1*	36	5.2 × 1.5 × 1	Polypectomy, and TH after recurrence	IB	Infiltrative	Diffuse, nested	Epithelioid cells	Present	Mild	3–5	Absent	Absent	Recurrence 14 m after polypectomy, NED (36 m) after TH
**Extrauterine metastasis cases**															
22	P	*ESR1::NCOA3*	66	6 × 4 × 3	THBSO	IB	Infiltrative	Sex cord, nested, adenosarcoma‐like	Epithelioid cells	Present	Mild	≤1	Absent	Absent	Multiple lung metastasis 58 m after THBSO, alive with disease until now (4 m)
	M	*ESR1::NCOA3*	71	Multiple lung metastasis	Lung biopsy	IVB	Infiltrative	Sex cord, nested	Epithelioid cells	Present	Mild	≤1	Absent	Absent	
23	P	*GREB1::NCOA1*	40	5.5 × 5 × 3	THBSO	IB	Infiltrative	Sex cord, diffuse	Epithelioid cells	Present	Moderate	≤1	Absent	Absent	Pelvic and omentum metastasis 157 m after THBSO, die of disease 4 m after recurrence
	M	*GREB1::NCOA1*	53	Distant metastasis	Local resection of metastatic lesions	IVB	Infiltrative	Sex cord, diffuse	Epithelioid cells	Present	Moderate	≤1	Absent	Absent	
24	P	*GREB1::NCOA2*	37	8 × 5 × 4	Myomectomy	IB	Infiltrative	Sex cord, diffuse, papillary, whorled	Spindle and epithelioid cells	Present	Moderate	3–5	Present	Absent	Pelvic multiple metastasis 12 m after myomectomy, and metastasis recurred 2 m after radical surgery, alive with disease until now (4 m)
	M	*GREB1::NCOA2*	38	Distant metastasis	THBSO and local resection of metastatic lesions	IVB	Infiltrative	Sex cord, diffuse	Epithelioid cells, rhabdoid cells	Present	Moderate	3–5	Absent	Present	
25	P	*ESR1::NCOA2*	26	5.5 × 3 × 2	polypectomy	IB	infiltrative	sex cord, diffuse, nested	epithelioid cells	present	mild	≤1	absent	absent	Uterine recurrence and pelvic lymph node metastasis 60 m after polypectomy, NED (14 m) after TH and pelvic lymph node dissection
	M	*ESR1::NCOA2*	31	9 × 5 × 5	TH and pelvic lymph node dissection	IIIC	Infiltrative	Sex cord, diffuse, nested	Epithelioid cells	Present	Mild	1–2	Present	Absent	

M, metastatic tumor; NED, no evidence of disease; P, primary tumor; TH, total hysterectomy; THBSO, total abdominal hysterectomy and bilateral salpingo‐oophorectomy.

The mean tumor size was 4.7 cm (range 1–15 cm), with 15 cases (60%) measuring ≤5 cm and 10 cases (40%) exceeding 5 cm. Notably all recurrent/metastatic cases (*n* = 6) occurred in tumors >5 cm. FIGO staging classified 15 patients (60%) as stage IA (≤5 cm) and 10 patients (40%) as stage IB (>5 cm). Surgical management included fertility‐preserving approaches (polypectomy/myomectomy in 14 cases) and radical hysterectomy [total hysterectomy (TH)/total hysterectomy bilateral salpingo‐oophorectomy (THBSO) in 11 cases].

**Figure 1:**
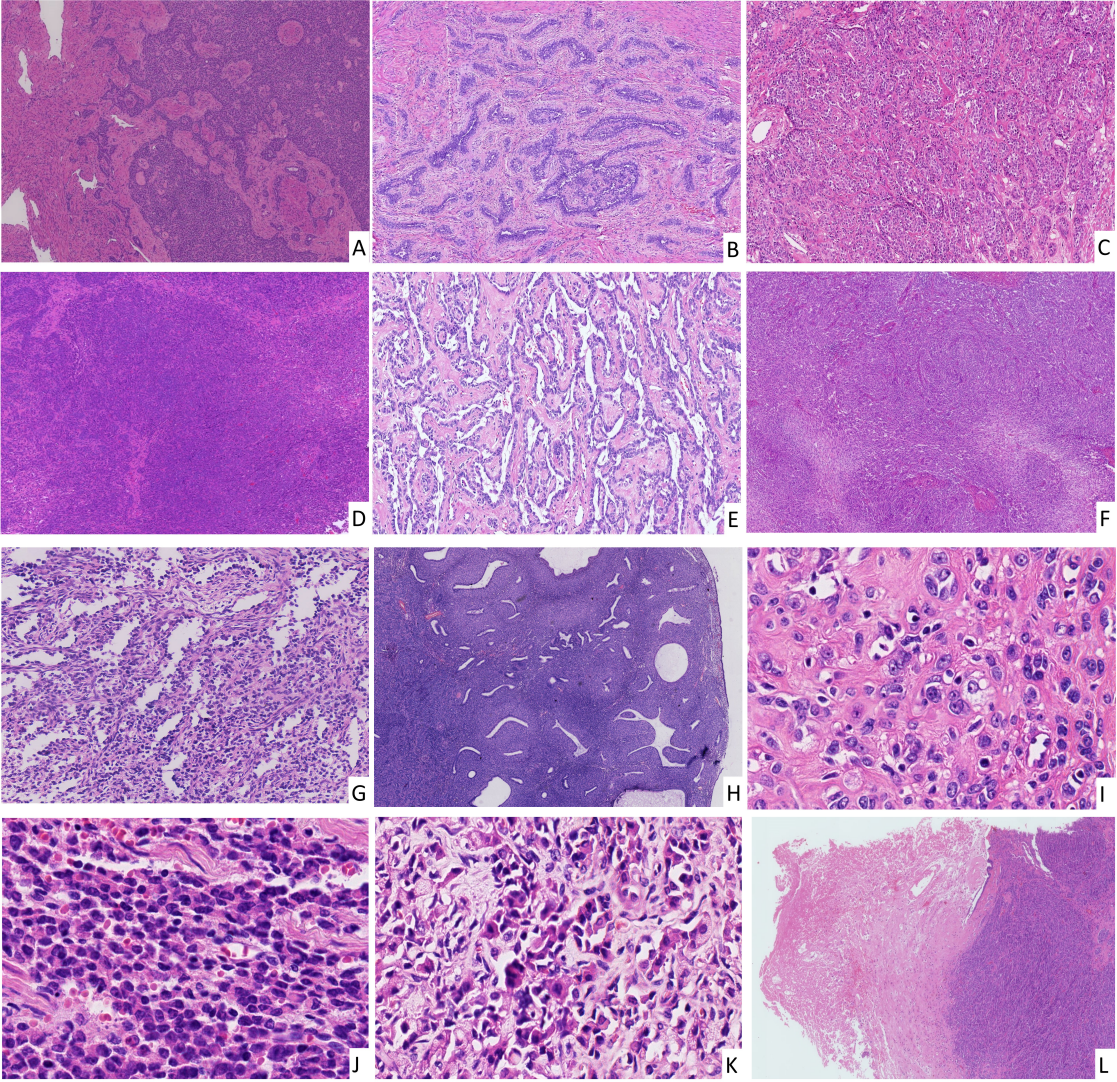
Common morphologic features of UTROSCTs. (A) Infiltrative edge (Case 8; H&E, ×4). (B) Sex cord‐like pattern (Case 6; H&E, ×4). (C) Nested pattern (Case 10; H&E, ×4). (D) Diffuse pattern (Case 20; H&E, ×4). (E) Retiform patterns (Case 12; H&E, ×4). (F) Whorled pattern (Case 6; H&E, ×4). (G) Papillary/pseudopapillary structure (Case 14; H&E, ×4). (H) Adenosarcoma‐like structure (Case 5; H&E, ×2). (I) Most cases have a small to moderate amount of pale, eosinophilic cytoplasm and a distinct nucleolus as seen in Case 9 (H&E, ×40). (J) Some cases have indistinct nucleoli as seen in Case 4 (H&E, ×40). (K) Cells with rhabdoid features can be observed in Case 4 (H&E, ×40). (L) Necrosis was observed in case 20 (H&E, ×2).

Histopathological analysis revealed infiltrative margins (Figure 1A and supplementary material, Figure S1) in 22 tumors (88%) with 3 tumors (12%) exhibiting well‐circumscribed borders (all ≤5 cm). This distribution pattern aligns with prior studies demonstrating that most UTROSCTs display focal microscopic infiltration despite a grossly circumscribed appearance [7]. All cases demonstrated heterogeneous architectural patterns, combining two or more growth configurations (Figure 1B–H). Sex cord‐like structures appear to be the most prevalent growth pattern (23/25, 92%), followed closely by diffuse (22/25, 88%) and nested (17/25, 68%) arrangements. Occasionally, whorled (5/25, 20%), retiform (2/25, 8%), papillary/pseudopapillary (2/25, 8%), and adenosarcoma‐like structures (2/25, 8%) can also be identified.

**Figure 2:**
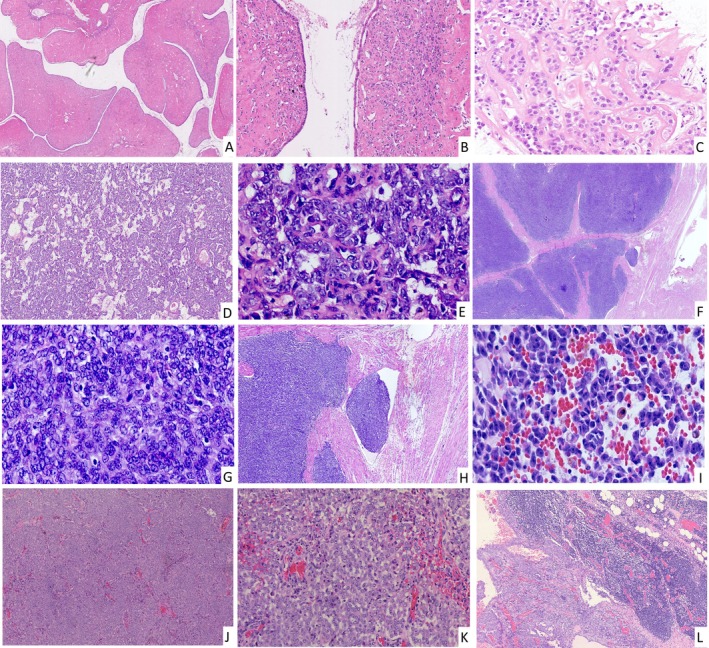
Morphologic features of UTROSCT cases with extrauterine metastasis. (A, B) The primary tumor of Case 22 exhibited an adenosarcoma‐like structure (A; H&E, ×2), with mild cellular atypia (B; H&E, ×10). (C) The lung biopsy of Case 22 revealed very mild cellular atypia at the metastatic site (H&E, ×40). (D) The primary tumor of Case 23 showed a sex cord‐like structure (H&E, ×4). (E) The omental metastasis of Case 23 showed moderate cellular atypia with infrequent mitotic figures (H&E, ×40). (F, G) The primary tumor of Case 24 showed a nodular and diffuse growth pattern (F; H&E, ×2), moderate cellular atypia with scarce mitosis (G; H&E, ×40). (H) LVSI was observed in Case 24 (H&E, ×10). (I) The tumor cells of the ovarian metastasis of Case 24 showed rhabdoid‐like features (H&E, ×40). (J) The primary tumor of Case 25 exhibited a diffuse growth pattern (H&E, ×4). (K) The tumor cells of Case 25 displayed mild cellular atypia, and mitosis was occasionally observed (H&E, ×40). (L) Pelvic lymph node metastasis was present in Case 25 (H&E, ×4).

Cytologically, tumors comprised spindle/epithelioid cells with variable nucleolar prominence (Figure 1I, J). Two cases (8%, Case 4 and the metastatic lesion of Case 24) demonstrated a rhabdoid appearance (Figures 1K, 2I). Cellular atypia was mild in 20 cases (80%; Figure 1I) and moderate in 5 (20%; Figure 2E, G). Mitotic activity was generally low (≤1/10 HPF in 16 cases; 64%) though one case exhibited 9–12 mitoses/10 HPF. LVSI was identified exclusively in two patients with extrauterine metastases (Figure 2H). Necrosis was present in four cases (16%; Figure 1L): one with extrauterine metastasis, one with uterine recurrence, and two achieved no evidence of disease (NED) by THBSO.

### Clinicopathologic characteristics of recurrent/metastatic cases

In the current study, recurrence or metastasis occurred in six cases (24%). Among these, two cases demonstrated localized uterine recurrence, while four patients exhibited extrauterine metastasis, with one case resulting in mortality (Table 1).

#### Uterine recurrence cases

Both uterine recurrences (Cases 20 and 21) presented with tumors exceeding 5 cm in diameter, characterized by diffuse/nested growth architectures and harboring *ESR1::NCOA3* and *GREB1::NCOA1* gene fusions. Initially managed with polypectomy, both patients experienced disease relapse at 6 and 14 months postoperatively, respectively. Subsequent TH effectively controlled the disease, with NED during 32–36 months of follow‐up.

#### Extrauterine metastasis cases

Case 22: A 6 cm uterine mass treated with THBSO recurred as multiple lung metastases after 5 years. Histologically, the tumor exhibited epithelioid cells forming adenosarcoma‐like structures with mild cellular atypia and rare mitotic figures (Figure 2A–C). Both primary and metastatic lesions harbored *ESR1::NCOA3* fusion, with *SMARCB1* heterozygous loss uniquely identified in metastases.

Case 23: A 40‐year‐old patient with a 5.5 cm primary tumor developed pelvic/omental metastases 13 years post‐THBSO. The tumor demonstrated diffuse and sex cord‐like growth patterns, moderate cellular atypia, and rare mitoses (Figure 2D, E). Shared *GREB1::NCOA1* fusion was detected, while *SMARCB1* loss occurred exclusively in metastases. The patient succumbed to disease progression 4 months post‐recurrence. Notably, Cases 22 and 23, which both demonstrated heterozygous *SMARCB1* loss, lacked rhabdoid features upon rigorous histopathological review.

**Figure 3:**
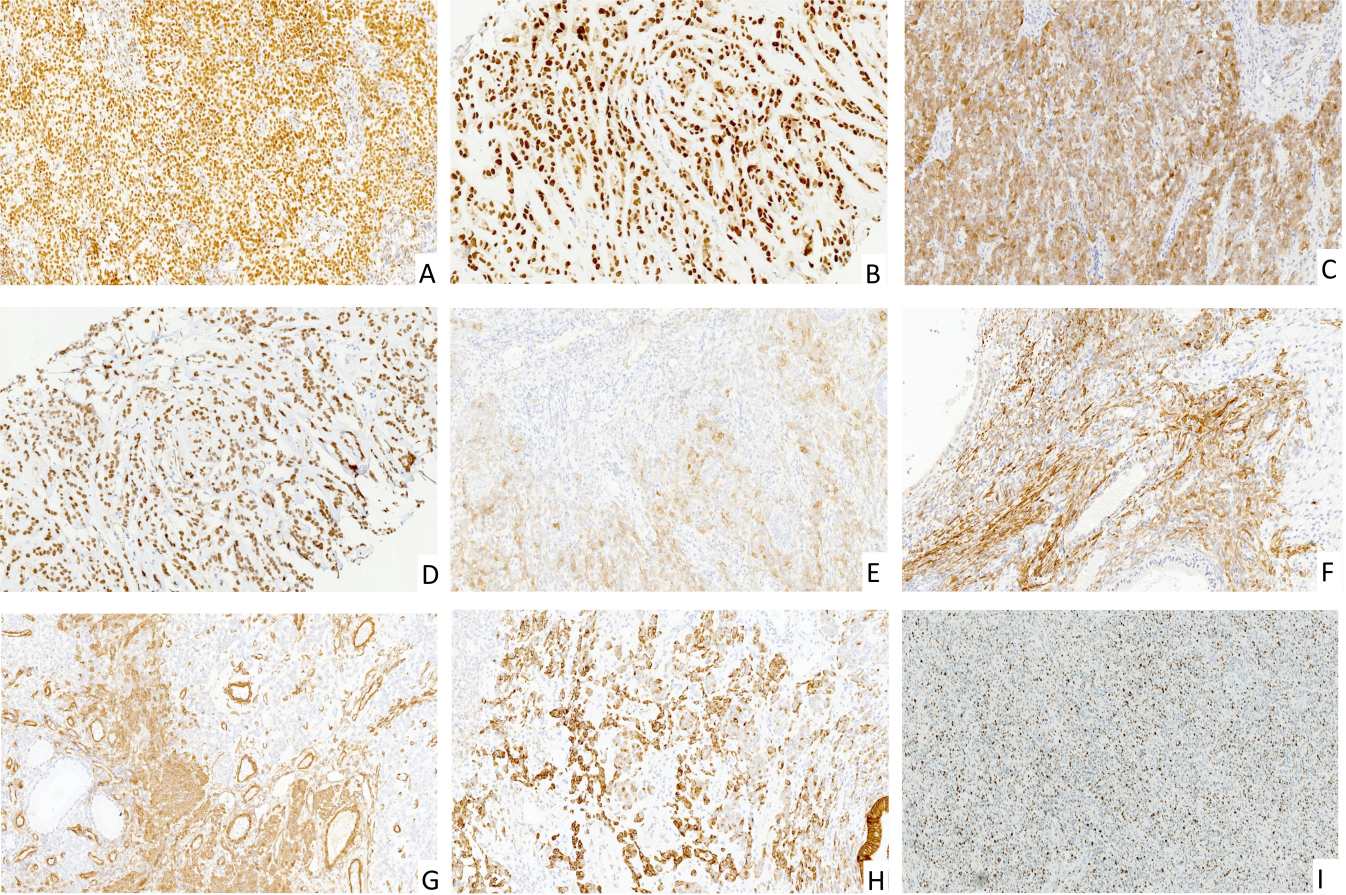
Representative immunohistochemistry patterns of UTROSCTs. All images are taken at ×100 magnification. (A) ER and (B) PR staining show diffuse expression of strong intensity. (C, D) Diffuse expression of sex cord‐stromal markers (C) Calretinin and (D) WT1. (E) Focal weak staining of endometrial stromal marker CD10. (F, G) Focal moderate to strong staining of smooth muscle markers, (F) Desmin and (G) SMA. (H) Focal strong expression of epithelial marker CK. (I) Ki67 staining in Case 18 shows around 50% positivity in the hot spot area.

**Figure 4:**
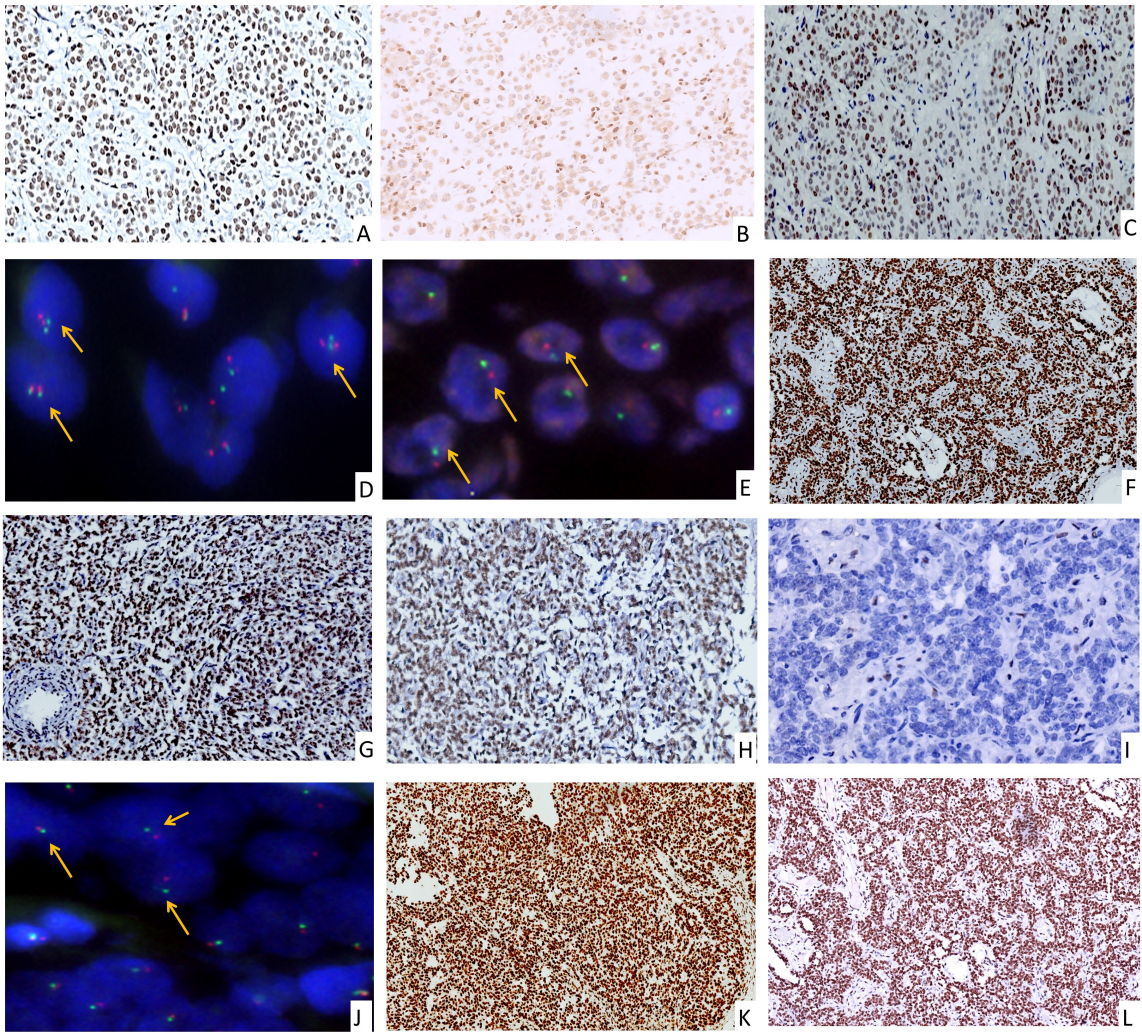
Representative immunohistochemistry (IHC) and FISH analysis of UTROSCTs with metastasis. All IHC images were captured at ×100 magnification. (A, B) INI‐1 staining in Case 22 reveals (A) diffuse strong staining in the primary tumor, with (B) markedly attenuated staining in the metastatic tumor; the strong expression in blood vessel epithelium and immune cells serves as an internal control. (C) BRG1 staining demonstrates diffuse positivity in Case 22. (D) FISH for *SMARCB1* (red) and *EWSR1* (green) in the primary tumor of Case 22 demonstrates the presence of two alleles of *SMARCB1* in each cell (arrow). (E) FISH of the metastatic tumor of Case 22 indicates the loss of one allele of both *SMARCB1* and *EWSR1* genes in each cell (arrow). (F, G) INI‐1 staining of Case 23 demonstrates diffuse strong expression in both (F) primary and (G) metastatic tumor. (H, I) BRG‐1 staining in Case 23 shows (H) diffuse strong staining in the primary tumor, and (I) abolished expression in the metastatic tumor. (J) FISH of the metastatic tumor of Case 23 reveals the loss of one allele of both *SMARCB1* and *EWSR1* genes in each cell (arrow). (K, L) ATRX staining in Case 24 demonstrates strong positivity in both (K) the primary tumor and (L) metastatic tumor.

Case 24: A 37‐year‐old patient initially treated with myomectomy for an 8‐cm uterine neoplasm developed pelvic and abdominal metastases within 1 year. Despite subsequent radical intervention comprising THBSO and metastatic tumor resection, the disease exhibited aggressive recurrence within 2 months post‐treatment. Histopathologic examination of the primary tumor revealed moderate nuclear atypia with occasional mitotic figures (3–5/10 HPF; Figure 2F, G). LVSI was identified within the myometrium (Figure 2H). Metastatic ovarian deposits exhibited a rhabdoid appearance (Figure 2I). Both lesions harbored *GREB1::NCOA2* fusion, with *ATRX* heterozygous loss restricted to metastases.

Case 25: A 26‐year‐old patient undergoing fertility‐sparing polypectomy for a 5.5‐cm tumor developed a 9‐cm recurrent uterine mass with LVSI and pelvic lymph node metastasis (Figure 2L and supplementary material, Figure S2) after 5 years. Subsequent TH with lymph node dissection achieved NED during 14 months of surveillance. The tumor displayed heterogeneous growth patterns (sex cord, diffuse, nested) with minimal atypia (Figure 2J, K). Molecular analysis identified *ESR1::NCOA2* fusion without additional DNA alterations.

### Immunohistochemical characteristic of UTROSCT

Table 2 comprehensively details the IHC analysis, revealing marked heterogeneity in the expression patterns of hormone receptors, lineage‐specific markers, and chromatin remodeling proteins across the tumor cohort.

**Table 2:** Immunohistochemistry of UTROSCT cases

Case no.		Fusion gene	Hormone receptors		Sex cord‐stromal markers				Endometrial stromal and smooth muscle markers				Epithelial markers	Chromatin remodeling proteins				Proliferation marker
			ER	PR	α‐inhibin	Calretinin	WT‐1	SF‐1	CD10	Desmin	SMA	h‐caldesmon	CKpan	INI‐1	BRG‐1	BRM	ATRX	Ki‐67
**Cases without recurrence/metastasis**																		
1		*ESR1::NCOA3*	+	+	+	+	Focal+	−	−	−	−	−	+	+	+	+	+	3%
2		*ESR1::NCOA3*	+	Weak+	Focal+	Focal+	Weak+	+	−	−	−	−	Focal+	+	+	+	+	<1%
3		*ESR1::NCOA3*	Weak+	+	−	+	Focal+	−	−	−	−	−	Focal+	+	+	+	+	10%
4		*ESR1::NCOA3*	Focal+	+	−	+	+	Focal+	Focal+	Focal+	−	−	Focal+	+	+	+	+	2%
5		*ESR1::NCOA3*	+	+	+	+	+	−	−	Focal+	−	−	+	+	+	+	+	10%
6		*ESR1::NCOA3*	+	+	Focal+	Focal+	+	−	Focal+	−	−	−	+	+	+	+	+	2%
7		*ESR1::NCOA3*	+	+	−	Focal+	Focal+	+	−	Focal+	Focal+	Focal+	+	+	+	+	+	3%
8		*ESR1::NCOA3*	+	Focal+	−	−	−	Focal+	Focal+	+	−	−	+	+	+	+	+	20%
9		*ESR1::NCOA3*	+	+	−	Focal+	Weak+	−	+	−	−	−	+	+	+	+	+	1%
10		*ESR1::NCOA3*	+	+	Focal+	+	Focal+	−	−	+	−	−	Focal+	+	+	+	+	2%
11		*ESR1::NCOA2*	+	+	+	Focal+	+	+	Focal+	Focal+	+	Focal+	+	+	+	+	+	10%
12		*ESR1::NCOA2*	+	+	Focal+	+	+	−	Focal+	Focal+	−	Focal+	+	+	+	+	+	3%
13		*GREB1::NCOA1*	+	+	−	Focal+	+	−	+	−	−	−	+	+	+	+	+	5–10%
14		*GREB1::NCOA1*	+	+	−	+	+	−	−	+	Focal+	−	+	+	+	+	+	20%
15		*GREB1::NCOA1*	+	+	Focal+	+	+	−	Focal+	−	−	−	+	+	+	+	+	15%
16		*GREB1::NCOA1*	+	+	Focal+	Focal+	Weak+	−	Weak+	+	+	Focal+	Focal+	+	+	+	+	20–30%
17		*GREB1::NCOA2*	+	+	+	+	+	Focal+	−	Focal+	Focal+	−	+	+	+	+	+	20%
18		*GREB1::SS18*	+	+	Focal+	−	−	−	−	−	Focal+	+	−	+	+	+	+	50%
19		*GREB1::CTNNB1*	+	+	−	Focal+	−	Focal+	−	Focal+	−	−	Focal+	+	+	+	+	40%
**Uterine recurrence cases**																		
20		*ESR1::NCOA3*	+	+	Focal+	Focal+	Focal+	−	Focal+	Focal+	Focal+	Focal+	Focal+	+	+	+	+	40%
21		*GREB1::NCOA1*	+	+	−	Focal+	+	Focal+	Focal+	−	−	−	Focal+	+	+	+	+	5%
**Extrauterine metastasis cases**																		
22	P	*ESR1::NCOA3*	+	+	−	Focal+	+	−	−	−	−	−	+	+	+	+	+	10%
	M	*ESR1::NCOA3*	+	+	−	Focal+	Focal+	−	−	−	−	−	+	Weak+	+	+	+	5–10%
23	P	*GREB1::NCOA1*	+	+	−	+	+	−	−	−	−	−	−	+	+	+	+	5%
	M	*GREB1::NCOA1*	+	+	−	+	Focal+	−	−	−	−	−	−	+	−	+	+	5%
24	P	*GREB1::NCOA2*	+	+	Focal+	Focal+	Focal+	Focal+	Focal+	Weak+	Focal+	Focal+	Focal+	+	+	+	+	30%
	M	*GREB1::NCOA2*	+	+	Focal+	Focal+	+	−	−	+	Focal+	−	+	+	+	+	+	40%
25	P	*ESR1::NCOA2*	+	+	+	+	+	+	−	Focal+	Focal+	Focal+	−	+	+	+	+	10%
	M	*ESR1::NCOA2*	+	+	+	+	+	+	−	Focal+	Focal+	Focal+	−	+	+	+	+	10%

‘+’, positive staining in ≥50% of the tumor cells; ‘−’, positive staining in <5% of tumor cells; ‘Focal+’, positive staining in 5–50% of the tumor cells; M, metastatic tumor; P, primary tumor.

Hormone receptor profiling demonstrated ubiquitous ER/PR positivity (Figure 3A, B), albeit with focal or weak staining intensity in a subset of cases, reinforcing the hormone‐dependent nature of these neoplasms.

Lineage‐specific differentiation markers showed variable distribution profiles (Figure 3C–H): All tumors exhibited at least focal positivity for one sex cord‐stromal marker, with Calretinin (92%, 23/25) and WT1 (88%, 22/25) being most prevalent, followed by α‐inhibin (60%, 15/25) and SF‐1 (40%, 10/25). Endometrial stromal marker CD10 was predominantly negative or focal positive, while smooth muscle markers displayed focal moderate expression in part of the cases, with Desmin (56%, 14/25) > SMA (36%, 9/25) > h‐Caldesmon (32%, 8/25). Epithelial differentiation was evident in 88% (22/25) of cases *via* cytokeratin expression. Proliferative activity, as assessed by Ki‐67, ranged from 1% to 50%, with 68% (17/25) of tumors showing low proliferation indices (≤10%) and four cases demonstrating exceptionally high activity (40–50%; Figure 3I).

Chromatin remodeling protein analysis revealed consistent robust expression of SWI/SNF complex subunits INI1, BRG1, and BRM across all tumor samples, with notable exceptions in Cases 22 and 23. In Case 22, primary tumor tissues retained strong INI1 staining, whereas metastatic deposits exhibited marked reduction in expression (Figure 4A, B). This differential expression correlated with *SMARCB1* heterozygous deletion detected in metastatic foci but not primary lesions. Conversely, Case 23 demonstrated preserved INI1 expression in both primary and metastatic tumors despite *SMARCB1* heterozygous loss in the latter (Figure 4F, G). Notably, metastatic sites displayed complete ablation of BRG1 immunostaining (Figure 4H, I), yet no somatic mutations or CNVs were identified in the *SMARCA4* gene (Table 3). These findings suggest potential disruptions in SWI/SNF complex integrity, aligning with prior reports indicating that a subset of *SMARCB1*‐driven neoplasms may retain INI1 expression while selectively losing BRG1 [16, 17, 18, 19, 20].

**Table 3:** Molecular alterations of UTROSCT cases

Case no.		Fusion gene	Detection method	Mutation (frequency)	Copy number variation	
					Amplification (ratio)	Deletion (ratio)
**Cases without recurrence/metastasis**						
1		*ESR1::NCOA3*	FISH and DNA‐based NGS	RAD51B (38.9%)	None	None
2		*ESR1::NCOA3*	FISH	\	\	\
3		*ESR1::NCOA3*	FISH	\	\	\
4		*ESR1::NCOA3*	FISH	\	\	\
5		*ESR1::NCOA3*	FISH and DNA‐based NGS	None	None	None
6		*ESR1::NCOA3*	FISH and DNA‐based NGS	None	None	None
7		*ESR1::NCOA3*	FISH	\	\	\
8		*ESR1::NCOA3*	FISH and DNA‐based NGS	None	None	WRN (0.6), RAD51 (0.6), FANCA (0.6)
9		*ESR1::NCOA3*	FISH	\	\	\
10		*ESR1::NCOA3*	RNA based NGS	\	\	\
11		*ESR1::NCOA2*	RNA based NGS	\	\	\
12		*ESR1::NCOA2*	RNA based NGS	\	\	\
13		*GREB1::NCOA1*	FISH	\	\	\
14		*GREB1::NCOA1*	FISH	\	\	\
15		*GREB1::NCOA1*	FISH and DNA‐based NGS	None	None	CHEK1 (0.6), MRE11A (0.6), ATM (0.6)
16		*GREB1::NCOA1*	FISH and DNA‐based NGS	None	MCL1 (3.1), CDK4 (2.1), ESR1 (2.1)	RAD54L (0.6), PTCH2 (0.5)
17		*GREB1::NCOA2*	FISH and DNA‐based NGS	None	None	NF1 (0.6), FANCA (0.5), JAK1 (0.5)
18		*GREB1::SS18*	RNA based NGS	\	\	\
19		*GREB1::CTNNB1*	RNA and DNA based NGS	None	None	None
**Uterine recurrence cases**						
20	Primary	*ESR1::NCOA3*	RNA and DNA‐based NGS	None	None	RAD51B (0.5)
	Recurrent	*ESR1::NCOA3*	RNA and DNA‐based NGS	None	None	RAD51B (0.6)
21	Primary	*\*	FISH and DNA‐based NGS		\	\
	Recurrent	*GREB1::NCOA1*	FISH and DNA‐based NGS	None	None	None
**Extrauterine metastasis cases**						
22	Primary	*ESR1::NCOA3*	FISH and DNA‐based NGS	SPEN (26.4%), KRAS (8.3%)	None	None
	Metastatic	*ESR1::NCOA3*	RNA and DNA‐based NGS	SPEN (12.7)	None	SMARCB1 (0.6), EP300 (0.7), CDH1 (0.6), CHEK2 (0.6), PTCH2 (0.6), FANCA (0.6), RAD54L (0.6), NF2 (0.6), ARID1A (0.6), JAK1 (0.6)
23	Primary	*GREB1::NCOA1*	RNA and DNA based NGS	MLL3 (9.6%)	None	RAD51D (0.7), RAD54L (0.6), PTCH2 (0.6), JAK1 (0.6)
	Metastatic	*GREB1::NCOA1*	RNA and DNA based NGS	MLL3 (7.9%)	Myc (1.7)	SMARCB1 (0.5), ARID1A (0.7), PTCH2 (0.6), RAD54L (0.6), EP300 (0.6), NF2 (0.6), CHEK2 (0.6)
24	Primary	*GREB1::NCOA2*	RNA and DNA based NGS	None	None	None
	Metastatic	*GREB1::NCOA2*	RNA and DNA based NGS	None	None	ATRX (0.5)
25	Primary	*ESR1::NCOA2*	RNA and DNA based NGS	None	None	None
	Metastatic	*ESR1::NCOA2*	RNA and DNA based NGS	None	None	None

In normal cells, the ratio of copy number is 1. \, not performed.

*ATRX*, a gene located on the X chromosome encoding a chromatin remodeling protein belonging to the SWI/SNF protein family [21], showed uniformly strong expression across all cases by IHC, including Case 24 where *ATRX* heterozygous deletion was identified exclusively in the metastatic tumor (Figure 4K, L). The paradoxical preservation of ATRX protein expression despite genomic loss warrants further mechanistic investigation, as discussed below.

### Molecular characterization of UTROSCT

#### Recurrent fusion gene landscape in UTROSCT

Integrative RNA sequencing and FISH analyses revealed six recurrent fusion types in our UTROSCT cohort (*n* = 25; Table 3). The fusion spectrum showed distinct prevalence: *ESR1::NCOA3* dominated (48%, 12/25), followed by *GREB1::NCOA1* (24%, 6/25), *ESR1::NCOA2* (12%, 3/25), *GREB1::NCOA2* (8%, 2/25), and single cases of *GREB1::SS18* and *GREB1::CTNNB1* (4% each). Clinical outcomes demonstrated disease recurrence in 24% (6/25) of patients: local recurrence (*n* = 2) occurred in *ESR1::NCOA3* and *GREB1::NCOA1* cases; and metastatic progression (*n* = 4) involved *ESR1::NCOA3*, *ESR1::NCOA2, GREB1::NCOA1*, and *GREB1::NCOA2* subtypes. Recurrence/metastasis rates varied by fusion subtype: *ESR1::NCOA3* (16.7%, 2/12), *GREB1::NCOA1* (33.3%, 2/6), *ESR1::NCOA2* (33.3%, 1/3), and *GREB1::NCOA2* (50%, 1/2). Notably, statistical evaluation showed no significant correlation between fusion partner identity (*ESR1* versus *GREB1*) and recurrence/metastasis risk (Table 4).

**Table 4:** Comparison of clinicopathologic parameters and molecular alterations between UTROSCTs with and without recurrence/metastasis

	UTROSCTs without recurrence/metastasis (*n* = 19)	UTROSCTs with recurrence/metastasis (*n* = 6)	*p*1	UTROSCTs with metastasis (*n* = 4)	*p*2
Clinical parameters					
Age (mean)	44.6	37.3	0.345	42.2	0.604
>40	9	1		1	
≤40	10	5		3	
Size (mean)	4.2	6.1	**0.001**	6.2	**0.008**
>5 cm	4	6		4	
≤5 cm	15	0		0	
FIGO stage			**0.001**		**0.008**
IA	15	0		0	
IB	4	6		4	
Treatment			0.661		1
Polypectomy/myomectomy	10	4		2	
TH or THBSO	9	2		2	
Histopathologic features					
Growth pattern			0.789		0.973
Sex cord	19 (100%)	4 (66.7%)		4 (100%)	
Diffuse	17 (89.5%)	5 (83.3%)		3 (75%)	
Nested	13 (68.4%)	4 (66.7%)		2 (50%)	
Whorled	4 (21%)	1 (16.7%)		1 (25%)	
Retiform	2 (10.5%)	0		0	
Papillary	2 (10.5%)	1 (16.7%)		1 (25%)	
Adenosarcoma	1 (5%)	1 (16.7%)		1 (25%)	
Cellular atypia			0.07		0.125
Mild	17	3		2	
Moderate	2	3		2	
Necrosis			0.234		0.453
Present	2	2		1	
Absent	17	4		3	
LVSI			0.050		**0.024**
Present	0	2		2	
Absent	19	4		2	
Mean mitosis			0.606		1.000
≤3/10HPF	15	4		3	
>3/10HPF	4	2		1	
Ki67			1.000		1.000
≤10	13	4		3	
>10	6	2		1	
Molecular features					
Fusion gene			0.653		1.000
*ESR1* altered	12	3		2	
*GREB1* altered	7	3		2	
*SMARCB1* loss			0.5		**0.024**
Present	0	2		2	
Absent	19	4		2	

Note that for tumors with recurrence/metastasis, the clinicopathologic parameters of the primary tumors were used in the analysis. *p*1 represents the *p* value for the comparison between UTROSCTs without recurrence/metastasis and those with recurrence/metastasis. *p*2 represents the *p* value for the comparison between UTROSCTs without recurrence/metastasis and those specifically with metastasis. The bold values correspond to statistically significant results with *p* < 0.05.

TH, total hysterectomy; THBSO, total abdominal hysterectomy and bilateral salpingo‐oophorectomy.

#### Genomic alteration signatures reveal metastasis‐associated drivers

DNA‐based NGS conducted on a subset of cases (*n* = 14), including all four with metastasis, revealed distinct patterns of genomic alterations (Table 3).

Across non‐metastatic UTROSCTs (*n* = 10, including cases without recurrence and the two cases with local uterine recurrence), somatic mutations were rare, except for a frameshift mutation in *RAD51B* (p.M39Ifs*8) identified in one case. *RAD51B*, a tumor suppressor critical for homologous recombination repair (HRR), exhibited compromised function due to this truncation. CNVs were detected in 60% (6/10) of non‐metastatic tumors, predominantly copy number losses in HRR‐related genes (*RAD51B, RAD51, RAD54L, WRN, FANCA, CHEK1, MRE11A*, and *ATM*). These findings suggest that impairment of the HRR pathway is a common feature in UTROSCT pathogenesis but does not inherently correlate with aggressive clinical behavior.

In contrast, metastatic tumors showed CNVs affecting key tumor suppressor genes, other than HRR genes. Cases 22 and 23 displayed heterozygous loss of *SMARCB1* (22q11.2) exclusively in metastases, accompanied by contiguous deletions spanning *CHEK2* (22q12.1), *NF2* (22q12.2), and *EP300* (22q13.2), forming an extended 22q loss. FISH confirmed the extended chromosomal deletions in both cases (Figure 3D, E, J). Statistical analysis highlighted *SMARCB1* loss as a strong predictor of metastasis (Table 4). Case 24 presented with heterozygous loss of *ATRX* in metastases. Collectively, copy number losses of critical genes involved in chromatin remodeling, especially *SMARCB1* and *ATRX*, were observed in UTROSCTs with extrauterine metastasis, suggesting an important mechanism for tumor evolution and progression.

Cases 22 and 23 also exhibited heterozygous loss of *PTCH2*, a negative regulator of the Sonic Hedgehog (Shh) pathway. Loss of *PTCH2* may constitutively activate Shh signaling, driving proliferation. Additionally, concurrent losses of HRR genes (*RAD54L, CHEK2*, and *FANCA*) were identified in cases with metastasis.

Notably, Case 25, with uterine recurrence and pelvic lymph node metastasis, lacked detectable mutations or CNVs. However, this patient achieved disease‐free status post‐TH, contrasting with uncontrolled metastatic progression in other cases.

### Comparative analysis of cases with or without recurrence/metastasis

Clinical, histopathological and molecular analyses comparing UTROSCTs with and without recurrence/metastasis are summarized in Table 4. Key clinicopathological parameters, specifically tumor size, FIGO stage, and LVSI, showed significant associations with recurrence/metastasis outcomes. No statistically meaningful correlations emerged for age, surgical modality, growth pattern, cellular atypia, mitotic activity, Ki67 proliferation index, or necrosis.

Notably, recurrent/metastatic tumors were significantly larger, with all six recurrent/metastatic cases exceeding 5 cm in diameter. In contrast, only four cases (4/19, 21%) of non‐recurrent tumors measured >5 cm, with a statistically significant difference (Table 4). FIGO stages (IB) were also overrepresented in the recurrent/metastatic cohort. LVSI was present in two cases with extrauterine metastasis but absent in others, demonstrating a strong statistical link between LVSI and metastatic potential.

Surgical approach did not significantly impact overall prognosis (Table 4). However, it is worth noting that, for tumors >5 cm, uterine recurrence rates reached 66.7% following polypectomy, compared to 0% after TH/THBSO, except for cases with extrauterine metastasis. This suggests TH/THBSO may reduce recurrence risk for larger tumors by ensuring complete resection, whereas polypectomy may leave residual disease due to size‐related technical challenges. Yet, even with THBSO, extrauterine metastasis occurred in 50% (2/4) of such cases, indicating metastasis mechanisms extend beyond surgical factors alone.

Histologically, UTROSCTs exhibited diverse growth patterns. Our analysis indicates no significant correlation between growth patterns and prognosis (Table 4). Certain histological characteristics, such as cellular atypia, necrosis, and mitosis, have been recognized as potential prognostic indicators in UTROSCT [9, 22]. Nevertheless, there exists a divergence of opinions regarding their prognostic significance [23, 24, 25, 26, 27]. In our cohort, cellular atypia was predominantly mild in the majority of cases, with only a minority (5/25, 20%) showing moderate atypia, and this did not demonstrate any statistical correlation with prognosis. Necrosis was observed in four cases, occurring both in those with and without recurrence, indicating no clear prognostic association. Mitosis was generally low in most cases, including those with metastasis, although it reached up to 9–12/10HPF in one case harboring the *GREB1::SS18* fusion gene but without recurrence. Based on our analysis, cellular atypia, necrosis, and mitosis did not emerge as reliable prognostic factors in UTROSCT.

Molecular profiling revealed distinct patterns associated with metastatic progression. Specifically, *ESR1* or *GREB1* fusion events demonstrated no discernible impact on clinical outcomes. In contrast, CNVs affecting genes involved in chromatin remodeling (e.g., *SMARCB1* and *ATRX*) were exclusively identified in metastatic tumors (Table 3). *SMARCB1* loss emerged as a strong predictor of metastatic progression, with significant correlations (Table 4).

## Discussion

UTROSCT, a rare neoplasm defined by sex cord‐like morphologic features and multi‐lineage immunophenotypic expression, typically follows a benign clinical course yet occasionally demonstrates recurrence or metastasis. Despite prior efforts to characterize prognostic risk factors, the unpredictable nature of UTROSCT – particularly in aggressive variants – persists due to its rarity. While recurrent fusion gene discoveries have advanced diagnostic specificity, the molecular determinants of variable clinical outcomes remain incompletely understood. Herein, we report integrated clinicopathological and molecular profiling of 25 UTROSCT cases to dissect prognostic drivers. Our analysis identifies significant associations between tumor size exceeding 5 cm, FIGO stage IB, LVSI, and adverse outcomes including recurrence and metastasis. Notably, we present novel evidence implicating CNVs in UTROSCT pathogenesis, specifically deletions targeting chromatin remodeling genes that correlate with metastatic propensity and poor prognosis. These findings expand the molecular taxonomy of UTROSCT and highlight CNV as a potential mechanism for metastatic progression in this enigmatic tumor.

Among 25 cases, six patients developed recurrence/metastasis – two with uterine recurrences and four exhibiting extrauterine metastases, including one fatality. This underscores the clinical significance of our findings, as UTROSCT typically demonstrates benign behavior with metastatic dissemination being exceptionally rare. Clinicopathological evaluation confirmed tumor size exceeding 5 cm and FIGO stage IB as adverse prognostic indicators, with all recurrent/metastatic cases>5 cm. This aligns with prior literature emphasizing tumor size as a critical determinant of outcome [9, 22]. LVSI, a well‐established prognostic marker across malignancies indicating heightened metastatic potential [28, 29], emerged as a robust predictor of metastasis in our cohort, particularly in cases involving pelvic lymph node compromise or multisite spread. While surgical modality did not significantly alter overall prognosis, TH or THBSO reduced uterine recurrence risk for tumors >5 cm compared to local resection *via* polypectomy. Notably, however, 50% (2/4) of extrauterine metastases occurred irrespective of surgical approach, suggesting intricate metastatic mechanisms operating beyond local disease control.

Histologically, UTROSCT displayed remarkable architectural diversity, with sex cord‐like, diffuse, and nested growth patterns predominating. Adenosarcoma‐like foci were identified in 8% of cases, warranting diagnostic vigilance due to their potential to mimic other entities, though these foci consistently coexisted with characteristic UTROSCT patterns. This wide spectrum of growth patterns aligns with prior reports and underscores a major diagnostic challenge – distinguishing UTROSCT from histologically similar neoplasms. While no direct correlation between specific growth patterns and clinical outcomes was observed, recognition of combinatorial architectural features may improve diagnostic precision. These findings emphasize the importance of integrating morphologic assessment with immunohistochemical and molecular studies for accurate UTROSCT classification.

Immunophenotypic profiling revealed consistent ER and PR expression, and variable expression of sex cord, smooth muscle and epithelial markers. Proliferative activity, as determined by the Ki‐67 index, was predominantly low across the study cohort – with Ki‐67 indices <10% observed in 68% of cases. This finding aligns with a prior meta‐analysis of 181 UTROSCTs, which reported a mean Ki‐67 labeling index of 8.7% [30]. Consistent with prior studies [7, 31], the Ki‐67 index demonstrated no significant prognostic value in this context. Collectively, these findings illustrate UTROSCT's multilineage immunophenotype, with no single immunohistochemical marker demonstrating sufficient specificity or sensitivity for definitive diagnosis. Instead, a combinatorial approach utilizing at least two markers from each lineage is recommended to enhance diagnostic accuracy and should be incorporated into routine diagnostic protocols.

Molecular analysis revealed that, while fusion gene subtypes did not significantly impact prognosis, CNV profiling identified distinct genomic events critical to metastatic evolution. Specifically, deletions targeting chromatin remodeling genes emerged as key drivers of aggressive behavior. Prior studies suggested *GREB1*‐rearranged UTROSCTs may carry elevated recurrence/metastasis risk [11], but our data showed comparable recurrence rates between *ESR1*‐altered (20%, 3/15) and *GREB1*‐altered (30%, 3/10) tumors, with fusion gene status not reaching prognostic significance.

Integrated genomic characterization of matched primary and metastatic lesions elucidated critical molecular alterations. Two metastatic tumors (Cases 22 and 23) harbored heterozygous *SMARCB1* deletions accompanied by broad 22q losses affecting *CHEK2, NF2*, and *EP300*. These structural variations were validated by FISH and correlated with reduced INI1 protein expression in Case 22. Notably, Case 23 presented a paradoxical phenotype: while maintaining genomic integrity of *SMARCA4*, this metastatic lesion demonstrated complete BRG1 protein loss coupled with preserved INI1 expression. Additionally, a metastatic tumor (Case 24) exhibited heterozygous *ATRX* copy number loss despite preserved ATRX protein expression. These findings highlight the role of chromatin regulatory gene deletions in UTROSCT progression and the need for integrated molecular‐protein analysis to fully capture their clinical implications.

The *SMARCB1* gene encodes the INI1 protein, a core subunit of the SWI/SNF chromatin remodeling complex. Loss of *SMARCB1* function destabilizes the SWI/SNF complex, impairing its chromatin occupancy and ability to repress target promoters, thereby promoting oncogenic transcriptional programs [32, 33]. *SMARCB1* deficiency occurs in up to 20% of human cancers [14, 34]; although the majority of *SMARCB1* deficient tumors harbor homozygous loss of *SMARCB1*, numerous papers have reported a subset of these tumors exhibiting heterozygous deletions or extended 22q losses [35, 36, 37]. Partial INI1 loss detected by IHC correlates with poor prognosis across multiple malignancies, including colorectal, pancreatic, and uterine carcinomas [38, 39, 40, 41, 42]. Notably, some *SMARCB1*‐driven tumors retain INI1 expression while losing BRG1 [16, 17, 18, 19, 20]. These findings underscore the complex functional interplay within the SWI/SNF chromatin remodeling machinery. The heterozygous loss of *SMARCB1* might result in a functional disruption of the SWI/SNF complex, which may drive oncogenic transformation through aberrant epigenetic reprogramming [43]. In our cohort, both patients with *SMARCB1* deletion experienced aggressive clinical courses, underscoring the prognostic implications of SWI/SNF disruption. However, while homozygous *SMARCB1* loss is well‐characterized in tumorigenesis, the clinical impact of heterozygous loss remains less defined. The observed heterozygous deletions in this study may represent passenger events, therapy‐induced changes, or sampling biases – hypotheses requiring validation in future prospective studies.

The *ATRX* gene, located on the X chromosome, encodes a chromatin remodeling protein belonging to the SWI/SNF family [21]. Functioning as a tumor suppressor, ATRX regulates chromatin architecture and mediates critical protein‐DNA interactions essential for genomic stability [44, 45]. While *ATRX* mutations or copy number alterations occur in approximately 6% of tumors across diverse lineages [46], their role in UTROSCT pathogenesis remains underexplored. In our study, heterozygous *ATRX* copy number loss was exclusively identified in the metastatic tumor of Case 24, notably without corresponding protein expression loss. This observation is particularly significant in female patients, who harbor two *ATRX* alleles but exhibit random X‐chromosome inactivation (XCI) during each cell division [47]. ATRX plays a critical role in maintaining XCI patterns [48], and heterozygous *ATRX* knockout in murine models disrupts XCI in extraembryonic tissues [49]. Extrapolating this finding, the heterozygous loss of *ATRX* in Case 24 may abrogate XCI, which might explain the retained expression of ATRX protein in the metastatic tumor. Defective XCI represents a well‐established oncogenic mechanism in breast and colorectal cancers [47, 50], suggesting a comparable role in UTROSCT metastasis. Remarkably, this *ATRX* copy number loss was the sole DNA alteration detected in the metastatic tumor, underscoring its potential as a driver of disease progression in this case. However, most *ATRX*‐deficient tumors harbored *ATRX* homozygous loss or inactivation mutations according to the literature; the functional implications of heterozygous *ATRX* loss specifically in female patients remain poorly characterized and necessitate systematic investigation.

Genomic profiling revealed copy number losses and mutations affecting HRR pathway genes in 57% (8/14) of the UTROSCT cases tested, distributed across both recurrent/metastatic and non‐recurrent tumors. This high frequency of HRR gene alterations suggests a fundamental role for DNA repair impairment in UTROSCT pathogenesis. Notably, however, these molecular changes did not correlate with adverse clinical outcomes. Collectively, these data elucidate a dual role for CNVs in UTROSCTs: (1) dysregulation of chromatin remodeling genes *via SMARCB1* and *ATRX* deletions strongly associates with metastatic potential and poor outcomes, while (2) HRR impairments, though implicated in tumorigenesis, show limited correlation with aggressive behaviors in this cohort.

A principal limitation of this study is the limited sample size, encompassing only four metastatic cases, which necessitates cautious interpretation of the findings. To ensure diagnostic rigor and sample homogeneity, we exclusively enrolled fusion‐positive UTROSCT cases. This selection criterion may restrict the generalizability of our results to morphologically typical yet fusion‐negative UTROSCTs. Consequently, the identification of CNVs as potential drivers of metastatic progression in UTROSCTs requires further validation. This validation should be conducted through multi‐institutional studies incorporating both fusion‐positive and fusion‐negative cohorts to enhance the representativeness of the findings and account for potential molecular heterogeneity. Despite these limitations, our analysis represents a critical contribution to ongoing efforts aimed at elucidating the molecular determinants underlying metastatic progression in UTROSCTs.

## Author contributions statement

JY contributed to methodology, investigation, formal analysis and writing the original draft. JZ, JL, YL, YW and AH acquired resources. CL contributed to funding acquisition, resources, supervision, writing, review and editing.

## Supporting information


**Figure S1.** Gross and microscopic images of Cases 7 and 24
**Figure S2.** Lymph node metastasis in Case 25
**Table S1.** Gene list of DNA‐based next‐generation sequencing
**Table S2.** Gene list of RNA‐based sequencing

## Data Availability

The authors confirm that the data supporting the findings of this study are available within the article and its supplementary materials.
