# Supplementary material for: Decoding UTROSCT heterogeneity: systematic clinicopathological evaluation combined with molecular profiling
Source: J Pathol Clin Res. 2025 Nov 29;12(1):e70055. doi: 10.1002/2056-4538.70055 (PMC12664526; doi:10.1002/2056-4538.70055)
Supplement: Supplementary file 1 — Figure S1. Gross and microscopic images of Cases 7 and 24 Figure S2. Lymph node metastasis in Case 25 Table S1. Gene list of DNA‐based next‐generation sequencing Table S2. Gene list of RNA‐based sequencing [file CJP2-12-e70055-s001.pdf]

Decoding UTROSCT heterogeneity: systematic clinicopathological evaluation combined with molecular profiling

J Yang *et al*. *J Pathol Clin Res* https://doi.org/10.1002/2056-4538.70055

Supplementary Figures S1 and S2
Supplementary Tables S1 and S2

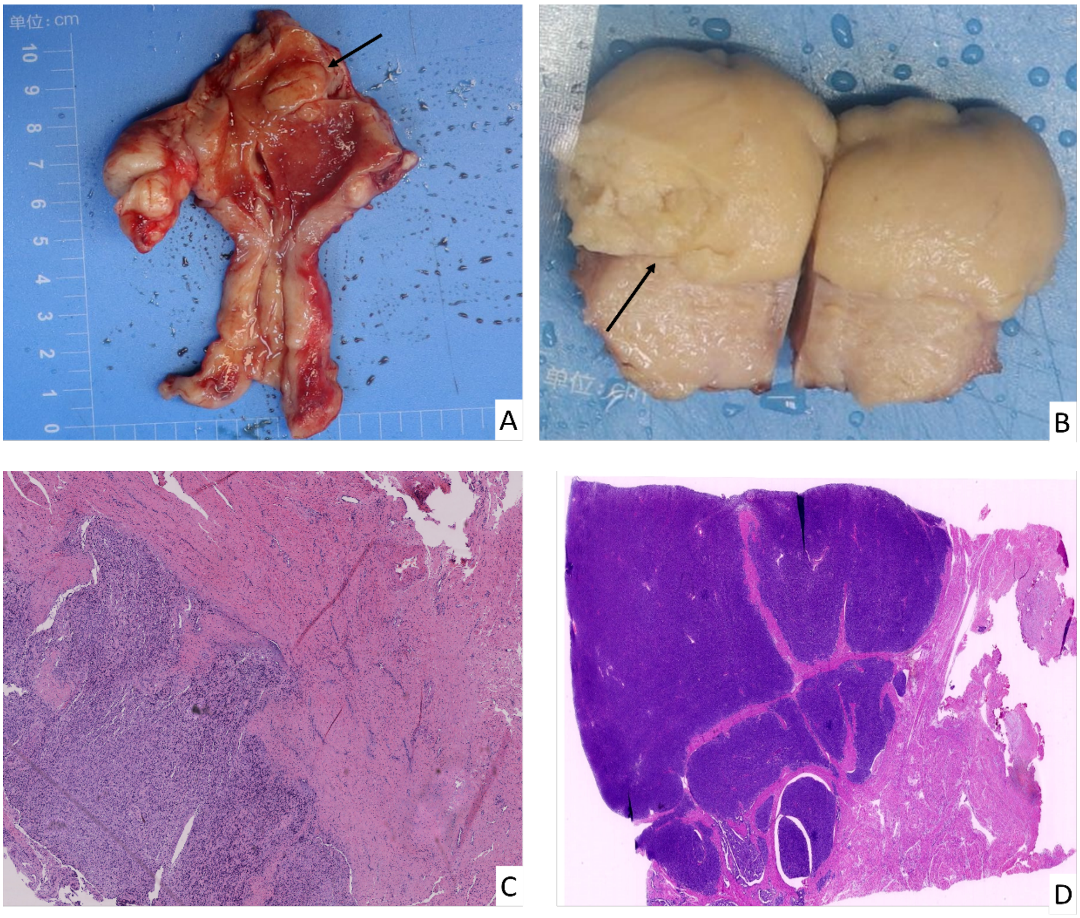

Figure S1. Gross and microscopic images of Cases 7 and 24.
(A) Gross image of Case 7 demonstrating a well-circumscribed uterine mass (arrow). (B) Gross image of Case 24 showing a uterine mass in a myomectomy specimen with irregular margins (arrow). (C, D) Microscopic images of Case 7 (C, H&E ×20) and Case 24 (D, H&E ×2) highlighting infiltrative tumor margins.

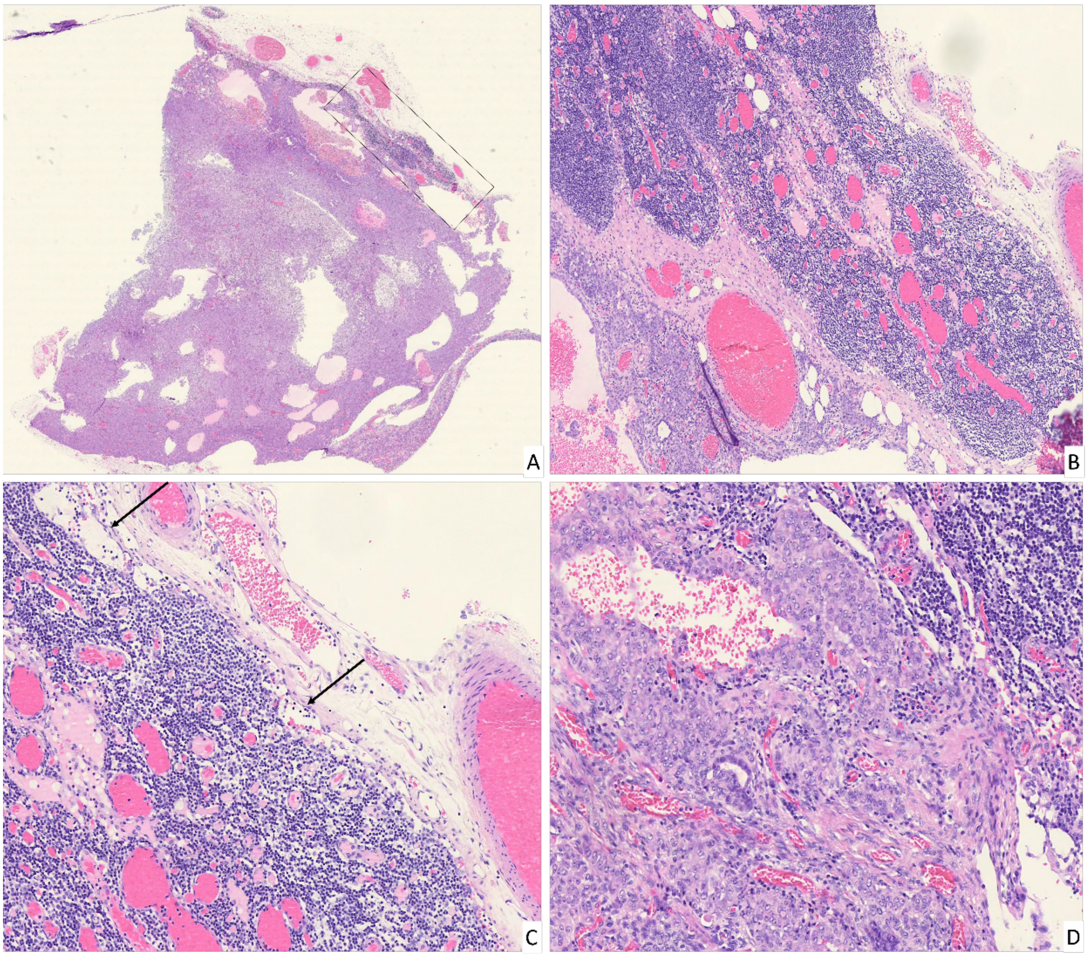

Figure S2. Lymph node metastasis in Case 25.

(A) Whole-slide panoramic image (H&E, ×1) of pelvic lymph node showing tumor metastasis. The majority of the lymph node is occupied by tumor cells, with focal residual lymph node architecture preserved in peripheral regions (enclosed within box). (B) Enlarged view (H&E, ×40) of the boxed area in (A), demonstrating adjacent residual lymphoid tissue (upper right) and infiltrating tumor cells (lower left). (C) High-power micrograph (H&E, ×200) highlighting residual lymph node structures, including capsule and subcapsular sinus (arrow). (D) High-magnification visualization (H&E, ×400) of metastatic tumor cells within the lymph node.

Table S1. Gene list of DNA-based next-generation sequencing.

1. The test covers all exons of 407 genes

ABL1	*ACVR1B*	*AKT1*	*AKT2*	*AKT3*	*ALK*	*ALOX12B*	*APC*	*AR*	*ARAF*
ARFRP1	*ARID1A*	*ARID1B*	*ARID2*	*ASXL1*	*ATM*	*ATR*	*ATRX*	*AURKA*	*AURKB*
AXIN1	*AXIN2*	*AXL*	*B2M*	*BACH1*	*BAP1*	*BARD1*	*BCL2*	*BCL2L1*	*BCL2L2*
BCL6	*BCOR*	*BCORL1*	*BLM*	*BMPR1A*	*BRAF*	*BRCA1*	*BRCA2*	*BRD4*	*BRIP1*
BTG1	*BTG2*	*BTK*	*C11orf30*	*CALR*	*CARD11*	*CASP8*	*CBFB*	*CBL*	*CCND1*
CCND2	*CCND3*	*CCNE1*	*CD22*	*CD274*	*CD70*	*CD79A*	*CD79B*	*CDC73*	*CDH1*
CDK12	*CDK4*	*CDK6*	*CDK8*	*CDKN1A*	*CDKN1B*	*CDKN2A*	*CDKN2B*	*CDKN2C*	*CEBPA*
CFTR	*CHD2*	*CHEK1*	*CHEK2*	*CIC*	*CREBBP*	*CRKL*	*CSF1R*	*CSF3R*	*CTCF*
CTNNA1	*CTNNB1*	*CUL3*	*CUL4A*	*CXCR4*	*CYLD*	*CYP17A1*	*DAXX*	*DDR1*	*DDR2*
DICER1	*DIS3*	*DNMT3A*	*DOT1L*	*EED*	*EGFR*	*EIF1AX*	*EP300*	*EPAS1*	*EPCAM*
EPHA2	*EPHA3*	*EPHA5*	*EPHA6*	*EPHB1*	*EPHB4*	*EPHB6*	*ERBB2*	*ERBB3*	*ERBB4*
ERCC1	*ERCC2*	*ERCC3*	*ERCC4*	*ERCC5*	*ERG*	*ERRFI1*	*ESR1*	*EXT1*	*EXT2*
EZH2	*FAM123B*	*FAM175A*	*FAM46C*	*FANCA*	*FANCC*	*FANCD2*	*FANCE*	*FANCF*	*FANCG*
FANCI	*FANCL*	*FANCM*	*FAS*	*FAT1*	*FAT2*	*FAT3*	*FBXW7*	*FGF10*	*FGF12*
FGF14	*FGF19*	*FGF23*	*FGF3*	*FGF4*	*FGF6*	*FGF7*	*FGFR1*	*FGFR2*	*FGFR3*
FGFR4	*FH*	*FLCN*	*FLT1*	*FLT3*	*FLT4*	*FOXA1*	*FOXL2*	*FOXP1*	*FUBP1*
GAB1	*GABRA6*	*GALNT12*	*GATA3*	*GATA4*	*GATA6*	*GEN1*	*GID4*	*GNA11*	*GNA13*
GNAQ	*GNAS*	*GRIN2A*	*GRM3*	*GSK3B*	*HDAC1*	*HDAC2*	*HGF*	*HNF1A*	*HOXB13*
HRAS	*HSD3B1*	*ID3*	*IDH1*	*IDH2*	*IFNG*	*IFNGR1*	*IGF1R*	*IKBKE*	*IKZF1*

IL7R	*INPP4B*	*IRF2*	*IRF4*	*IRS2*	*JAK1*	*JAK2*	*JAK3*	*JUN*	*KDM5A*
KDM5C	*KDM6A*	*KDR*	*KEAP1*	*KEL*	*KIT*	*KLHL6*	*KRAS*	*LRP1B*	*LTK*
LYN	*MAF*	*MAP2K1*	*MAP2K2*	*MAP2K4*	*MAP3K1*	*MAP3K13*	*MAPK1*	*MAX*	*MC1R*
MCL1	*MDM2*	*MDM4*	*MED12*	*MEF2B*	*MEN1*	*MERTK*	*MET*	*MITF*	*MKNK1*
MLH1	*MLH3*	*MLL*	*MLL2*	*MLL3*	*MPL*	*MRE11A*	*MS4A1*	*MSH2*	*MSH3*
MSH6	*MST1R*	*MTAP*	*MTOR*	*MUTYH*	*MYB*	*MYC*	*MYCL1*	*MYCN*	*MYD88*
NBN	*NCOR1*	*NF1*	*NF2*	*NFE2L2*	*NFKBIA*	*NKX2-1*	*NOTCH1*	*NOTCH2*	*NOTCH3*
NPM1	*NRAS*	*NSD1*	*NT5C2*	*NTHL1*	*NTRK1*	*NTRK2*	*NTRK3*	*P2RY8*	*PALB2*
PARK2	*PARP1*	*PARP2*	*PARP3*	*PAX5*	*PBRM1*	*PCK1*	*PDCD1*	*PDCD1LG2*	*PDGFRA*
PDGFRB	*PDK1*	*PIK3C2B*	*PIK3C2G*	*PIK3CA*	*PIK3CB*	*PIK3CD*	*PIK3CG*	*PIK3R1*	*PIK3R2*
PIM1	*PMS1*	*PMS2*	*POLD1*	*POLE*	*POT1*	*PPARG*	*PPM1D*	*PPP2R1A*	*PPP2R2A*
PRDM1	*PRKAR1A*	*PRKCI*	*PRSS1*	*PTCH1*	*PTCH2*	*PTEN*	*PTPN11*	*PTPRD*	*PTPRO*
QKI	*RAC1*	*RAD21*	*RAD50*	*RAD51*	*RAD51B*	*RAD51C*	*RAD51D*	*RAD52*	*RAD54L*
RAF1	*RARA*	*RB1*	*RBM10*	*RECQL*	*RECQL4*	*REL*	*RELA*	*RET*	*RHOA*
RICTOR	*RINT1*	*RNF43*	*ROS1*	*RPA1*	*RPL22*	*RPS20*	*RPTOR*	*RREB1*	*RSPO2*
RSPO3	*RUNX1*	*SDHA*	*SDHAF2*	*SDHB*	*SDHC*	*SDHD*	*SERPINB3*	*SERPINB4*	*SETD2*
SF3B1	*SGK1*	*SLX4*	*SMAD2*	*SMAD3*	*SMAD4*	*SMARCA4*	*SMARCB1*	*SMO*	*SNCAIP*
SOCS1	*SOX2*	*SOX9*	*SPEN*	*SPINK1*	*SPOP*	*SPTA1*	*SRC*	*SRSF2*	*STAG2*
STAT3	*STAT4*	*STK11*	*SUFU*	*SYK*	*TACSTD2*	*TBX3*	*TCF7L2*	*TEK*	*TERC*
TERT	*TET2*	*TGFBR2*	*TIPARP*	*TMEM127*	*TMPRSS2*	*TNFAIP3*	*TNFRSF14*	*TOP1*	*TOP2A*
TP53	*TSC1*	*TSC2*	*TSHR*	*TYRO3*	*U2AF1*	*VEGFA*	*VHL*	*WHSC1*	*WHSC1L1*

WRN	*WT1*	*XPO1*	*XRCC2*	*ZMAT3*	*ZNF217*	*ZNF703*	-	-	-

2. The test covers all the introns, promoters, and fusion and break regions of 49 genes

ALK	*ARHGAP26*	*BCL2L11*	*BRAF*	*BRCA1*	*BRD4*	*C15orf55*	*CLDN18*	*CD74*	*EGFR*
EML4	*ERG*	*ETV4*	*ETV5*	*ETV6*	*EWSR1*	*EZR*	*FGFR1*	*FGFR2*	*FGFR3*
KIF5B	*KIT*	*MAML2*	*MET*	*MSH2*	*MYB*	*MYC*	*MYCL1*	*NCOA4*	*NOTCH2*
NTRK1	*NTRK2*	*NTRK3*	*PDGFRA*	*PLEKHS1*	*PMS2*	*RAF1*	*RET*	*RELA*	*ROS1*
RSPO2	*RSPO3*	*SDC4*	*SLC34A2*	*TERT*	*TFE3*	*TMPRSS2*	*TPM3*	*YAP1*	-

3. The test covers part of the exons of 611 genes

ABCA13	*ABCB1*	*ABCC1*	*ABCC11*	*ABCC2*	*ABCG2*	*ABL2*	*ACACA*	*ACIN1*	*ACTB*
ACTG1	*ACTG2*	*ACVR2A*	*ACVRL1*	*ADAM29*	*ADAMTS5*	*ADCY1*	*AFF2*	*AFF3*	*AHNAK*
AKAP9	*ALB*	*AMOT*	*ANGPT1*	*ANK3*	*ANKRD11*	*ANKRD30A*	*ANKRD30B*	*APEX1*	*APOBEC3B*
ARAP3	*ARFGEF1*	*ARFGEF2*	*ARHGAP29*	*ARHGAP35*	*ARID4B*	*ARID5B*	*ARNT*	*ASCL4*	*ASH1L*
ASMTL	*ASPM*	*ASTN1*	*ASXL2*	*ATP12A*	*ATP1A1*	*ATP2B3*	*BAZ2B*	*BBC3*	*BBS9*
BCAS1	*BCL10*	*BCL11A*	*BCL11B*	*BCL2A1*	*BCL2L11*	*BCL3*	*BCL9*	*BCR*	*BIRC3*
BMPR2	*BNC2*	*BPTF*	*BRD2*	*BRD3*	*BRSK1*	*BRWD1*	*BUB1*	*C15orf23*	*C15orf55*
C1QA	*C1S*	*C3orf70*	*C7orf53*	*C8orf34*	*CACNA1E*	*CADM2*	*CAMTA1*	*CASP1*	*CASQ2*
CBLB	*CBR1*	*CBR3*	*CCDC168*	*CCNA1*	*CCNB3*	*CCT3*	*CCT5*	*CD33*	*CD5L*
CD74	*CDA*	*CDH11*	*CDH18*	*CDH23*	*CDK13*	*CHD1*	*CHD4*	*CHD6*	*CHD8*
CHD9	*CHFR*	*CHI3L1*	*CHN1*	*CIITA*	*CLDN18*	*CLP1*	*CNOT3*	*CNOT4*	*CNTN1*
CNTN5	*CNTNAP1*	*CNTNAP5*	*COL1A1*	*COL2A1*	*COL5A1*	*COL5A2*	*COL5A3*	*COPS2*	*CPS1*
CRIPAK	*CRLF2*	*CRNKL1*	*CRTC1*	*CSF1*	*CSMD1*	*CSMD3*	*CSNK1A1*	*CSNK1G3*	*CTLA4*

CTNNA2	*CTNND1*	*CUX1*	*CYBA*	*CYP19A1*	*CYP1A1*	*CYP1B1*	*CYP2A13*	*CYP2C8*	*CYP2D6*
CYP3A4	*DCC*	*DDX3X*	*DDX5*	*DEK*	*DHX35*	*DHX9*	*DIS3L2*	*DLC1*	*DMD*
DNAH6	*DNAJB1*	*DNM2*	*DNMT3B*	*DOCK2*	*DPYD*	*DRGX*	*DTX1*	*DUSP22*	*DYSF*
E2F3	*EBF1*	*ECT2L*	*EEF1A1*	*EGR3*	*EIF2AK3*	*EIF2C3*	*EIF3A*	*EIF4A2*	*EIF4G3*
ELAC2	*ELF1*	*ELF3*	*ELMO1*	*EME2*	*EMID2*	*EML4*	*EPC1*	*EPHA1*	*EPHA4*
EPHA7	*EPHB2*	*EPOR*	*EPPK1*	*EPS15*	*ESR2*	*ETS1*	*ETV1*	*ETV5*	*ETV6*
EWSR1	*EZR*	*F8*	*FAM131B*	*FAM135B*	*FAM157B*	*FAM5C*	*FAP*	*FASLG*	*FAT4*
FCGR1A	*FCGR2A*	*FCGR2B*	*FCGR3A*	*FCRL4*	*FLG*	*FLI1*	*FLNC*	*FMN2*	*FN1*
FNDC4	*FOXA2*	*FOXO1*	*FOXQ1*	*FRMPD4*	*FUS*	*FXR1*	*FZD1*	*G3BP2*	*GAB2*
GATA1	*GATA2*	*GFRAL*	*GIGYF1*	*GKN2*	*GLB1L3*	*GLI1*	*GLI2*	*GLI3*	*GNG2*
GPC3	*GPR124*	*GPS2*	*GPX1*	*GRB7*	*GSTM5*	*GSTP1*	*GUSB*	*H3F3A*	*H3F3B*
H3F3C	*HCLS1*	*HCN1*	*HDAC4*	*HDAC9*	*HECW1*	*HEY1*	*HIST1H1C*	*HIST1H1D*	*HIST1H1E*
HIST1H2AC	*HIST1H2AG*	*HIST1H2AL*	*HIST1H2AM*	*HIST1H2BC*	*HIST1H2BD*	*HIST1H2BJ*	*HIST1H2BK*	*HIST1H2BO*	*HIST1H3B*
HIST1H3C	*HIST1H3D*	*HIST1H3F*	*HIST1H3G*	*HIST1H3H*	*HIST1H3I*	*HIST1H4I*	*HIST3H3*	*HLA-A*	*HLA-B*
HLA-C	*HLF*	*HMCN1*	*HNF1B*	*HNRPDL*	*HOXA11*	*HOXA13*	*HOXA3*	*HOXA9*	*HOXC13*
HOXD11	*HOXD13*	*HSP90AA1*	*HSP90AB1*	*HSPA8*	*HSPD1*	*HSPH1*	*ICK*	*IFITM3*	*IGF1*
IGF2	*IGF2R*	*IGLL5*	*IKZF2*	*IKZF3*	*IL10*	*IL1RAPL1*	*IL21R*	*IL6*	*IL6ST*
IMPG1	*ING1*	*INHBA*	*INPP4A*	*INPPL1*	*INSR*	*IRF6*	*IRS1*	*ITGB3*	*ITK*
ITSN1	*KALRN*	*KAT6A*	*KAT6B*	*KCNJ5*	*KCNQ2*	*KDM2B*	*KIF5B*	*KLF4*	*KLK1*
KRTAP5-5	*L3MBTL1*	*LAMA2*	*LATS1*	*LATS2*	*LCP1*	*LEF1*	*LIFR*	*LPHN2*	*LPP*
LRP2	*LRP4*	*LRP5*	*LRP6*	*LRRC7*	*LRRK2*	*LZTS1*	*MAD1L1*	*MAGI2*	*MAML2*

MAML3	*MAPK3*	*MCC*	*MDC1*	*MECOM*	*MEF2C*	*MGA*	*MIB1*	*MIOS*	*MKL1*
MLL4	*MLLT3*	*MMP11*	*MMP2*	*MN1*	*MNDA*	*MNX1*	*MSH4*	*MSN*	*MSR1*
MTHFR	*MTRR*	*MYH11*	*MYH14*	*MYH9*	*MYO3A*	*MYOD1*	*NAP1L1*	*NAV3*	*NCAM2*
NCOA3	*NCOA4*	*NCOR2*	*NCSTN*	*NDUFA13*	*NFATC4*	*NFE2L3*	*NKX3-1*	*NLRC3*	*NOS3*
NOTCH4	*NQO1*	*NR1I2*	*NR2F2*	*NR4A2*	*NRG1*	*NRP2*	*NRXN1*	*NTM*	*NUMA1*
NUP210	*NUP93*	*NUP98*	*OBSCN*	*OMD*	*OPCML*	*OR11G2*	*OR2T4*	*OR4A15*	*OR4C6*
OR5L2	*OR6F1*	*P4HB*	*PABPC1*	*PABPC3*	*PAG1*	*PAK1*	*PAK3*	*PAK7*	*PASK*
PAX3	*PAX7*	*PC*	*PCDH18*	*PCSK6*	*PCSK7*	*PDE4DIP*	*PDGFB*	*PDILT*	*PER1*
PGR	*PHF6*	*PIK3C2A*	*PIK3C3*	*PKD1L2*	*PKHD1*	*PLAG1*	*PLCB1*	*PLCG1*	*PLCG2*
PLK1	*PLXNA1*	*PLXNB2*	*PNRC1*	*POLQ*	*POM121*	*POM121L12*	*PPP1R17*	*PPP6C*	*PRDM16*
PREX2	*PRF1*	*PRKAA1*	*PRKCB*	*PRKDC*	*PRRX1*	*PRX*	*PSG2*	*PSIP1*	*PSMB1*
PSMB5	*PTGS1*	*PTGS2*	*PTPN2*	*PTPRB*	*PTPRK*	*PTPRS*	*PTPRT*	*PTPRU*	*RAB35*
RAC2	*RAD54B*	*RANBP2*	*RASGRP1*	*RBL1*	*RELN*	*RFC1*	*RGS3*	*RHEB*	*RHOH*
RHOT1	*RIT1*	*RNASEL*	*ROBO1*	*ROBO2*	*ROBO3*	*ROCK1*	*RPGR*	*RPS6KB1*	*RPS6KB2*
RUNX1T1	*RUNX2*	*RXRA*	*RYR1*	*RYR2*	*SBDS*	*SCUBE2*	*SDC4*	*SEMA3A*	*SEMA3E*
SEMA6A	*SERPINA7*	*SETBP1*	*SETDB1*	*SF1*	*SGCZ*	*SH2B3*	*SH3PXD2A*	*SI*	*SIN3A*
SLC16A1	*SLC1A2*	*SLC22A16*	*SLC22A2*	*SLC22A3*	*SLC34A2*	*SLCO1B3*	*SLIT1*	*SLIT2*	*SMARCD1*
SMARCE1	*SMC1B*	*SNTG1*	*SNX29*	*SOD2*	*SOS1*	*SOX10*	*SOX17*	*SPRR3*	*SPSB4*
SRD5A2	*SRGAP3*	*SRSF7*	*STAG1*	*STAT1*	*SUCLG1*	*SULT1A1*	*SVEP1*	*SYNCRIP*	*SYNE1*
TAF1	*TAF15*	*TAF1L*	*TAL1*	*TBL1XR1*	*TBX15*	*TBX22*	*TCEB1*	*TCF12*	*TCF3*
TCF4	*TCL1A*	*TEC*	*TENM3*	*TET1*	*TFDP1*	*TFE3*	*TGFBR1*	*THBS2*	*TJP1*

TLL2	*TLR4*	*TLX3*	*TMEM132D*	*TNFSF11*	*TNN*	*TP53BP1*	*TP63*	*TP73*	*TPM3*
TPR	*TRAF2*	*TRAF7*	*TRIM58*	*TRIO*	*TRPC5*	*TRRAP*	*TSHZ2*	*TSHZ3*	*TTF1*
TUBA3C	*TUBB3*	*TUSC3*	*TXNIP*	*TYMS*	*TYR*	*UBE2D2*	*UBR5*	*UGT1A1*	*UMPS*
UPF3B	*USH2A*	*USP6*	*USP8*	*VEZF1*	*VIM*	*VTCN1*	*WASF3*	*WDTC1*	*WIPF1*
WNK1	*WNT5A*	*WSCD2*	*WWOX*	*WWP1*	*WWP2*	*XPC*	*XRCC1*	*XRCC3*	*YAP1*
ZBTB16	*ZC3H11A*	*ZFHX3*	*ZFP36L1*	*ZFP36L2*	*ZFPM2*	*ZIC3*	*ZNF521*	*ZNF638*	*ZNF750*
ZNF804B	-	-	-	-	-	-	-	-	-

Table S2. Gene list of RNA-based sequencing.

1. List of 555 genes

ABI1	*ABL1*	*ABL2*	*ACBD6*	*ACLY*	*ACSL3*	*ACSL6*	*ACTB*	*AFF1*	*AFF3*
AFF4	*AGK*	*AGPAT5*	*AGTRAP*	*AKAP13*	*AKAP9*	*AKT1*	*AKT2*	*AKT3*	*ALDH2*
ALK	*ANK3*	*AP3B1*	*ARAF*	*ARFIP1*	*ARHGAP26*	*ARHGEF12*	*ARID1A*	*ARNT*	*ASIC2*
ASPSCR1	*ASXL1*	*ATF1*	*ATG4C*	*ATG5*	*ATIC*	*ATP8B2*	*AXL*	*BAIAP2L1*	*BBS9*
BCL10	*BCL11A*	*BCL11B*	*BCL2*	*BCL3*	*BCL6*	*BCL7A*	*BCL9*	*BCOR*	*BCR*
BIRC3	*BRAF*	*BRCA1*	*BRCA2*	*BRD1*	*BRD3*	*BRD4*	*BTG1*	*C15orf55*	*C2orf44*
CAMTA1	*CANT1*	*CARD11*	*CARS*	*CASC5*	*CBFA2T3*	*CBFB*	*CBL*	*CCDC170*	*CCDC6*
CCER1	*CCNB1IP1*	*CCNB3*	*CCND1*	*CCND2*	*CCND3*	*CCNY*	*CD274*	*CD28*	*CD74*
CDH11	*CDK4*	*CDK6*	*CDX1*	*CDX2*	*CHCHD7*	*CHD1*	*CHIC2*	*CHN1*	*CHRM1*
CIC	*CIITA*	*CLCN6*	*CLP1*	*CLRN3*	*CLTC*	*CLTCL1*	*CNBP*	*CNTRL*	*COL1A1*
COX6C	*CREB1*	*CREB3L1*	*CREB3L2*	*CREBBP*	*CRLF2*	*CRTC1*	*CRTC3*	*CSF1*	*CSF1R*
CTAGE5	*CTLA4*	*CTNNB1*	*CUTA*	*CXCR7*	*CXorf67*	*CYP39A1*	*DAZL*	*DDIT3*	*DDX10*
DDX5	*DDX6*	*DEK*	*DNAJB1*	*DUSP22*	*DUX4*	*EBF1*	*EGFR*	*EHF*	*EIF3E*
EIF3K	*EIF4A2*	*ELF4*	*ELK4*	*ELL*	*ELN*	*EML4*	*EP300*	*EPC1*	*EPOR*
EPS15	*ERBB2*	*ERBB4*	*ERC1*	*ERG*	*ERO1L*	*ESR1*	*ESRP1*	*ETS1*	*ETV1*
ETV4	*ETV5*	*ETV6*	*EWSR1*	*EZH2*	*EZR*	*FAM114A2*	*FAM131B*	*FAM22A*	*FAM22B*
FAM86C1	*FBXL18*	*FBXO38*	*FCGR2B*	*FCHSD1*	*FCRL4*	*FERMT2*	*FEV*	*FGFR1*	*FGFR1OP*
FGFR2	*FGFR3*	*FGFR4*	*FHDC1*	*FHIT*	*FIP1L1*	*FKBP15*	*FLI1*	*FLJ27352*	*FLT1*
FLT3	*FN1*	*FNBP1*	*FOSB*	*FOXO1*	*FOXO3*	*FOXO4*	*FOXP1*	*FSTL3*	*FUS*
GAB1	*GAB2*	*GABBR2*	*GAS7*	*GEMIN2*	*GLI1*	*GLIS2*	*GMDS*	*GMPS*	*GNAI1*
GNAS	*GOLGA5*	*GOPC*	*GPBP1L1*	*GPHN*	*GREB1*	*GRHL2*	*GTF2IRD1*	*HAS2*	*HERPUD1*

HEY1	*HIP1*	*HIST1H4I*	*HLF*	*HMGA1*	*HMGA2*	*HMGN2P46*	*HN1*	*HOOK3*	*HOXA11*
HOXA13	*HOXA3*	*HOXA9*	*HOXC11*	*HOXC13*	*HOXD11*	*HOXD13*	*HSP90AA1*	*HSP90AB1*	*HSPA8*
IGF2BP3	*IGH*	*IGK*	*IGL*	*IKZF1*	*IKZF2*	*IKZF3*	*IL2*	*IL21R*	*IL3*
IL6R	*INTS4*	*IRF2BP2*	*IRF4*	*ITK*	*ITPR2*	*JAK1*	*JAK2*	*JAK3*	*JAZF1*
KAT6A	*KAT6B*	*KDM5A*	*KDR*	*KDSR*	*KIAA1549*	*KIF5B*	*KIT*	*KLC1*	*KLK2*
KRAS	*KTN1*	*LASP1*	*LCK*	*LCP1*	*LGR5*	*LHFP*	*LIFR*	*LMO1*	*LMO2*
LPP	*LRIG3*	*LTK*	*LTV1*	*LYL1*	*LYN*	*MACF1*	*MAF*	*MAFB*	*MALT1*
MAML3	*MAP2K2*	*MAP3K3*	*MAST1*	*MAST2*	*MBOAT2*	*MBTD1*	*MCPH1*	*MDM2*	*MDS2*
MAML2	*MEAF6*	*MECOM*	*MET*	*MGEA5*	*MITF*	*MKL1*	*MKRN1*	*MLF1*	*MLL*
MLLT1	*MLLT10*	*MLLT11*	*MLLT3*	*MLLT4*	*MLLT6*	*MN1*	*MNX1*	*MRC2*	*MSH2*
MSI2	*MSN*	*MTCP1*	*MUC1*	*MYB*	*MYC*	*MYCL1*	*MYD88*	*MYH11*	*MYH9*
NAB2	*NACA*	*NACC2*	*NBEAP1*	*NCKIPSD*	*NCOA1*	*NCOA2*	*NCOA3*	*NDRG1*	*NF1*
NFATC1	*NFATC2*	*NFIA*	*NFIB*	*NFIX*	*NFKB2*	*NIN*	*NKAIN2*	*NOTCH1*	*NOTCH2*
NOTCH3	*NPM1*	*NR4A3*	*NRG1*	*NSD1*	*NTN1*	*NTRK1*	*NTRK2*	*NTRK3*	*NUMA1*
NUMBL	*NUP107*	*NUP214*	*NUP98*	*OFD1*	*OLIG2*	*OMD*	*P2RY8*	*PACS1*	*PAFAH1B2*
PATZ1	*PAX3*	*PAX5*	*PAX7*	*PAX8*	*PBX1*	*PCM1*	*PCSK7*	*PDCD1LG2*	*PDE4DIP*
PDE8B	*PDGFB*	*PDGFRA*	*PDGFRB*	*PER1*	*PGR*	*PHF1*	*PICALM*	*PIK3CA*	*PIK3CD*
PIM1	*PKD1L1*	*PKN1*	*PLA2R1*	*PLAG1*	*PLXND1*	*PML*	*POR*	*POU2AF1*	*POU5F1*
PPAP2B	*PPARG*	*PPFIBP1*	*PPP1CB*	*PRCC*	*PRDM16*	*PRKACA*	*PRKAR2A*	*PRKCA*	*PRKCB*
PRKCE	*PRRX1*	*PSIP1*	*PTBP3*	*PTCH1*	*PTK7*	*PTPRK*	*QKI*	*RABEP1*	*RAD51B*
RAF1	*RALGDS*	*RANBP17*	*RANBP2*	*RAP1GDS1*	*RARA*	*RBM14*	*RBM15*	*RBMS1*	*RBPMS*
RELA	*RET*	*RGS22*	*RHOH*	*RMI2*	*RNF130*	*RNF213*	*RNF216*	*ROS1*	*RPL22*
RPN1	*RPS2P32*	*RPS6KB1*	*RRP15*	*RSPO2*	*RSPO3*	*RSU1P2*	*RUNX1*	*RUNX1T1*	*RUNX2*

SDC4	*SEC16A*	*SEC31A*	*SEPT5*	*SEPT6*	*SEPT8*	*SEPT9*	*SET*	*SETBP1*	*SFPQ*
SH3GL1	*SLC1A2*	*SLC22A1*	*SLC26A6*	*SLC34A2*	*SLC45A3*	*SMARCA5*	*SMO*	*SND1*	*SNX29*
SP3	*SPECC1*	*SPECC1L*	*SQSTM1*	*SRGAP3*	*SRSF3*	*SS18*	*SS18L1*	*SSBP2*	*SSH2*
SSX1	*SSX2*	*SSX4*	*STAT5B*	*STAT6*	*STIL*	*STL*	*STRN*	*SUSD1*	*SUZ12*
SVOPL	*SYCP1*	*SYK*	*TACC1*	*TACC2*	*TACC3*	*TADA2A*	*TAF15*	*TAF3*	*TAL1*
TAL2	*TBCEL*	*TBL1XR1*	*TCEA1*	*TCF12*	*TCF3*	*TCF7L2*	*TCL1A*	*TCL6*	*TEC*
TECTA	*TERT*	*TET1*	*TFE3*	*TFEB*	*TFG*	*TFPT*	*TFRC*	*TG*	*THADA*
TLX1	*TLX3*	*TMCC1*	*TMPRSS2*	*TNFRSF11A*	*TNFRSF17*	*TOP1*	*TP53*	*TP63*	*TPM3*
TPM4	*TPR*	*TRIM24*	*TRIM27*	*TRIM33*	*TRIP11*	*TRPS1*	*TTL*	*TYK2*	*UACA*
UBE2L3	*USH1G*	*USP6*	*VAV1*	*VCL*	*VGLL2*	*VTI1A*	*WHSC1*	*WHSC1L1*	*WIF1*
WT1	*WWTR1*	*XPO1*	*YAP1*	*YPEL5*	*YWHAE*	*YY1*	*ZBTB16*	*ZC3H7B*	*ZMYM2*
ZNF384	*ZNF444*	*ZNF521*	*ZNF700*	*ZNF703*					

2. Rearrangement of special genes

MET Exon14 skipping	*EGFR* VIII	*ALK* KDD	*BRAF* KDD	*EGFR* KDD	*ERBB2* KDD	*ERBB4* KDD
FGFR1 KDD	*FGFR2* KDD	*FGFR3* KDD	*FGFR4* KDD	*FLT3* KDD	*KIT* KDD	*MET* KDD
NTRK1 KDD	*NTRK2* KDD	*PDGFRA* KDD	*PDGFRB* KDD	*RET* KDD	*ROS1* KDD	*PIK3CA* KDD
NTRK3 KDD	*TMPRSS2* KDD					
